# Assessment of guided lateral maxillary sinus lift procedure with simultaneous implant placement using stereolithographic surgical guide: a randomized controlled clinical study

**DOI:** 10.1007/s10006-025-01399-3

**Published:** 2025-05-17

**Authors:** Wael Hussein, Ahmed Younis, Ahmad Elrawdy, Mohamed ElSholkamy

**Affiliations:** 1 Faculty of Dentistry, Misr International University, KM 28 Cairo – Ismailia Road, Ahmed Orabi District, Cairo, 6363001 Egypt; 2 Faculty of Dentistry, Suez Canal University, 4.5 Km the Ring Road, Ismailia, 8366004 Egypt

**Keywords:** Stereolithography, Computer guided, Lateral window, Ppen sinus elevation, Dental implants, Maxillary sinus

## Abstract

**Aim:**

The aim of this study is to assess the efficacy of the stereolithographic surgical guide in reducing intraoperative and postoperative complication during lateral sinus lift operation.

**Materials and methods:**

A parallel randomized controlled prospective clinical study was conducted on fourteen patients requiring thirty dental implants in the posterior maxilla and diagnosed with reduced vertical bone height. Lateral Maxillary Sinus Lift procedure with simultaneous implant placement was performed to all patients. Stereolithographic surgical guides for lateral window osteotomy and implant drilling and placement were used in the study group, while lateral osteotomy and implant drilling and placement was done freehand in the control group. A cone beam computed tomography was taken immediately and six months post-sinus lifting. Intraoperative and postoperative complications were assessed, pain and edema were assessed using visual analogue scale and vertical bone was assessed using fusion module of cone beam computed tomography.

**Results:**

All dental implants demonstrated high survival rates with no statistically significant difference observed in intraoperative or postoperative complications. In terms of new vertical bone gain, both groups exhibited satisfactory and successful outcomes. Concerning pain, there was no statistically significant difference between the two groups except after two days, the study group showed statistically significantly lower pain score than the control group. While regarding the severity of edema, the study group showed statistically significantly higher prevalence of moderate and severe edema than control group which showed higher prevalence of mild edema.

**Conclusion:**

According to the current study it has been concluded that there was no remarkable difference between the outcomes of both methods.

The study protocol and its consent form were approved by the ethical committee of Suez Canal University (No.432/2021); and registered retrospectively on 23 April 2024 on PACTR (PACTR20240875463218) (pactr.samrc.ac.za/TrialDisplay.aspx?TrialID = 30442).

## Introduction

Maxillary atrophy is common in edentulous patients and complicates the restoration of posterior maxilla, leading to problems with the traditional restorations’ retention. The use of dental implants has become a favorable option over the use of traditional prosthetic restorations. Functional and aesthetic rehabilitation of the atrophic maxillary posterior region requires adequate bone for the long-term stability and survival rate of dental implants. [1, 2]

If the vertical height of the remaining bone is not sufficient for implant placement, the maxillary sinus must be elevated to allow for implant placement. The deficient vertical bone height is not just a limitation in fully edentulous patients but can be seen in partially edentulous patients. This is due to sinus pneumatization following the extraction of teeth. Maxillary sinus elevation followed by bone grafting using autogenous bone or bone substitutes allows for sufficient vertical bone height for implant placement. [3]

Tatum [4] in 1976 first described the use of a lateral maxillary sinus lift to raise the maxillary sinus floor and this technique was first published by Boyne and James [5] in 1980. During sinus floor elevation, the space created between the residual maxillary ridge and the elevated Schneiderian membrane is usually filled with graft material. The grafted bone will allow reliable implant placement, either simultaneously with the sinus lift procedure, if the available bone will result in acceptable implant primary stability, or as a second stage after the graft site matures [6–8]. The research done in the last forty years has shown that implant placement combined with sinus floor elevation is a predictable treatment modality, and the implant survival rate is comparable to traditional implant placement protocols [9, 10].

However, common intraoperative complications associated with lateral sinus lift include sinus (Schneiderian) membrane perforation, wound dehiscence, infection, excessive bleeding, and loss of graft material [11, 12]. It is important to avoid abnormal sinus anatomy (such as the sinus septum and arteries) during surgical planning, as they can cause a higher incidence of intraoperative complications such as membrane perforation and bleeding [13–15].

Proper and accurate design and location of the lateral side window is necessary to facilitate the sinus lifting process and help the surgeon to successfully lift the sinus membrane. In addition, direct visualization helps in better packing of the entire graft space, which is essential to provide primary stability for the simultaneous insertion of the implants with the sinus elevation procedure [16]. Clinical evidence also shows that in the case of the minimum residual alveolar bone height, simultaneous implant placement and open sinus elevation is a viable treatment modality without compromising the primary stability of the implant [17].

The application of digital technology has proven to be a valuable tool for diagnosis and treatment planning. Data obtained from CBCT scans, digital oral scans, and facial scans can be integrated and manipulated using special computer software, providing surgeons with an interactive interface to perform virtual surgery planning before the actual surgery [18, 19]. The use of three-dimensional printing technology can reproduce the patient bone anatomy into a physical model, as well as precise surgical templates and guides [20, 21]. The use of a three-dimensional model prior to sinus surgery can simulate the surgical procedure to improve the sinus augmentation technique and outcomes [22].

The first time the manufacture of three-dimensional printed surgical guides for outlining the lateral windows was done by Mandelaris & Rosenfeld in 2008 [23]. Several authors have modified the technique by effectively using various implant planning software [24–26].

The precision of stereolithographic guides may mitigate risks associated with anatomical variations in atrophic maxillae, potentially reducing membrane perforation and optimizing lateral window osteotomy and implant placement accuracy which are key factors this study evaluates. While stereolithographic guides are increasingly used in sinus lift procedures, comparative evidence of their efficacy in reducing intraoperative complications versus conventional techniques remains limited, particularly in cases of severe maxillary atrophy.

This study aimed to assess the efficacy of the stereolithographic surgical guide in reducing intraoperative and postoperative complications during the lateral sinus lift procedure.

## Methods

### Study design

The hypothesis of this study is that that stereolithographic surgical guides will significantly reduce both intraoperative complications and postoperative complications compared to conventional freehand techniques during lateral sinus lift procedure.

The study design is interventional prospective randomized clinical trials. The unit of analysis and randomization is the individual implant site. In the study group the lateral window osteotomy of the lateral maxillary sinus lift was done using stereolithographic surgical guide with simultaneous implant placement through the same guide. While in the control group both the lateral window osteotomy and implants were placed freehand.

The study protocol and its consent form were approved by the ethical committee of Suez Canal University (No.432/2021); and was registered retrospectively on 23 April 2024 on PACTR (PACTR20240875463218) (pactr.samrc.ac.za/TrialDisplay.aspx?TrialID = 30442). All patients signed informed consent and authorized the use of their data for research. All procedures conducted followed the Helsinki Declaration in 1964.

### Selection criteria

Patients included in this study were selected from those seeking implant placement to restore their missing maxillary posterior teeth with insufficient vertical ridge height. The study was conducted in the outpatient clinic of the Oral and Maxillofacial Department, Faculty of Dentistry, Suez Canal University.

### Blinding

Although patients were not blinded to treatment allocation, all outcome assessors and statisticians were masked to group assignment during data collection and analysis. The surgeon performing the procedures could not be blinded because of the use of the stereolithographic surgical guide in the study group and therefore the surgeon had to be aware of it.

### Sample size calculation

Sample size determination was based upon using implant stability using resonance frequency analysis (RFA) as the Primary outcome. The effect size (f) based on the results of Bechara et al. (2016) [27] was 71 ÷ 0.5(SD) for the test group and 71.6 for the control group. Using alpha level of 0.05 (5%) and B level of 0.20 (20%) i.e. power = 80%; the estimated minimum required sample size (n) was 22 cases (11 per group). Over-sampling was performed to compensate for 15% drop-out rate. The number was increased to a total sample size of 30 (15 in each group).

### Eligibility and criteria

#### Inclusion criteria:

- Males and females ≥ 18 years of age.
- ASA I and ASA II.
- Patients having partial edentulism in the posterior region of the maxilla.
- Edentulous sites consist of native non augmented bone.
- Horizontal ridge dimension minimum of 5 mm
- The vertical ridge dimension 4–7 mm.
- Bone quality of D2 or D3.
- Enough inter-arch distance.

#### Exclusion criteria:

- Patients with active acute infection or residual lesion related to the edentulous sites.
- Acute maxillary sinus pathosis
- Remaining root dislodged in the Maxillary sinus.
- Patients that lack a stable occlusion or have parafunctional habits.
- Patients with poor oral hygiene who are not amenable to motivation and improvement.
- Smokers who smoke more than ten cigarettes a day.
- Pregnant or lactating mothers.
- Alcohol and drug abuse.
- Treatment with radiation therapy in the craniofacial region within the previous 12 months.

### Grouping

Implant Sites were randomly allocated to either one of the two groups using random.org.

**Group 1**: Lateral window was done using stereolithographic surgical guide for placement of fifteen implants in posterior edentulous maxillary regions. (**Study group**).

**Group 2**: Lateral window was done conventionally (freehand) for placement of fifteen implants in posterior edentulous maxillary regions. (**Control group**).

**Fig. 1:**
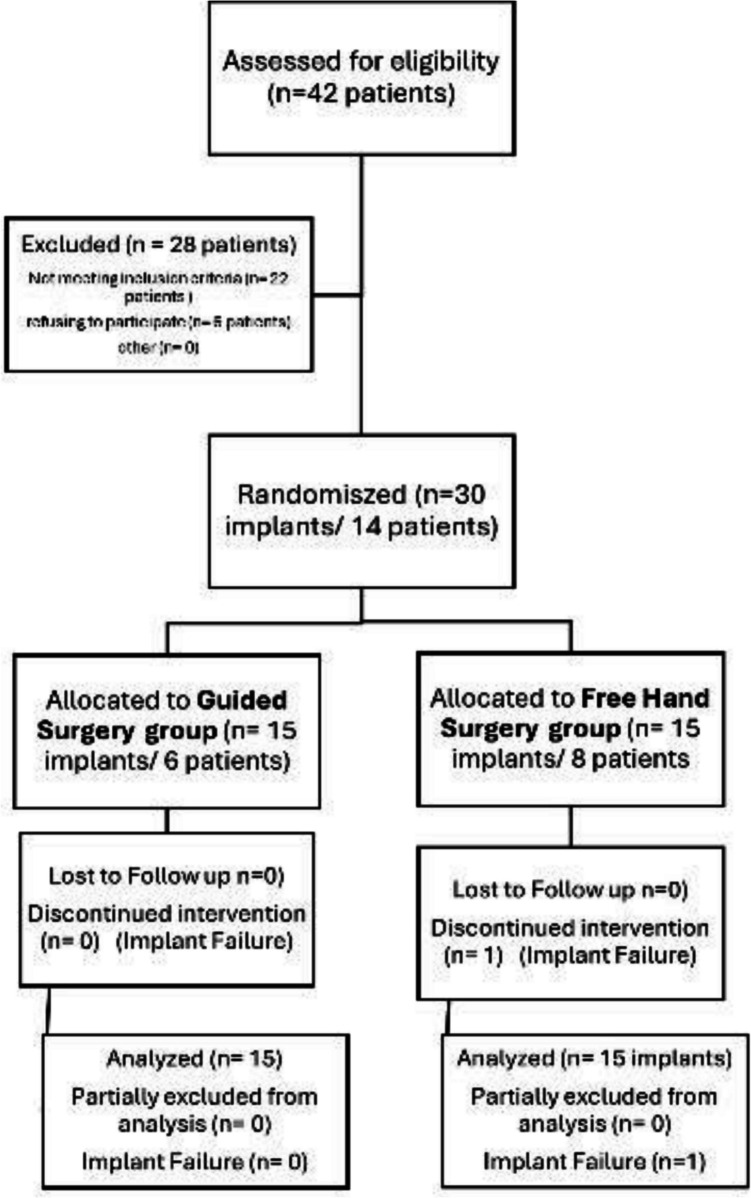
CONSORT diagram showing the flow of participants through each stage of the randomized trial

According to the CONSORT flowchart in [Fig. 1], participant recruitment and follow-up procedures were detailed.

### Pre-surgical preparation

After thorough diagnosis and the patient was considered eligible for this study, Cone Beam Computed Tomography (CBCT), with large field of view to scan the whole maxillary sinus starting from the orbital floor to the maxilla, was taken for the patient using Scanora three-dimensional imaging system(Scanora three-dimensional imaging system (CBCT machine): Sordex, Helsinki, Finland).

Radiographic Examination was conducted to measure the available bone height, width, and relative density. The presence of bony septa and/or anastomosis between the posterior superior artery and infraorbital artery were also noted during this examination.

To imitate the natural dentition impressions were taken and a study cast was poured using dental stone.

### For study group

**Fig. 2:**
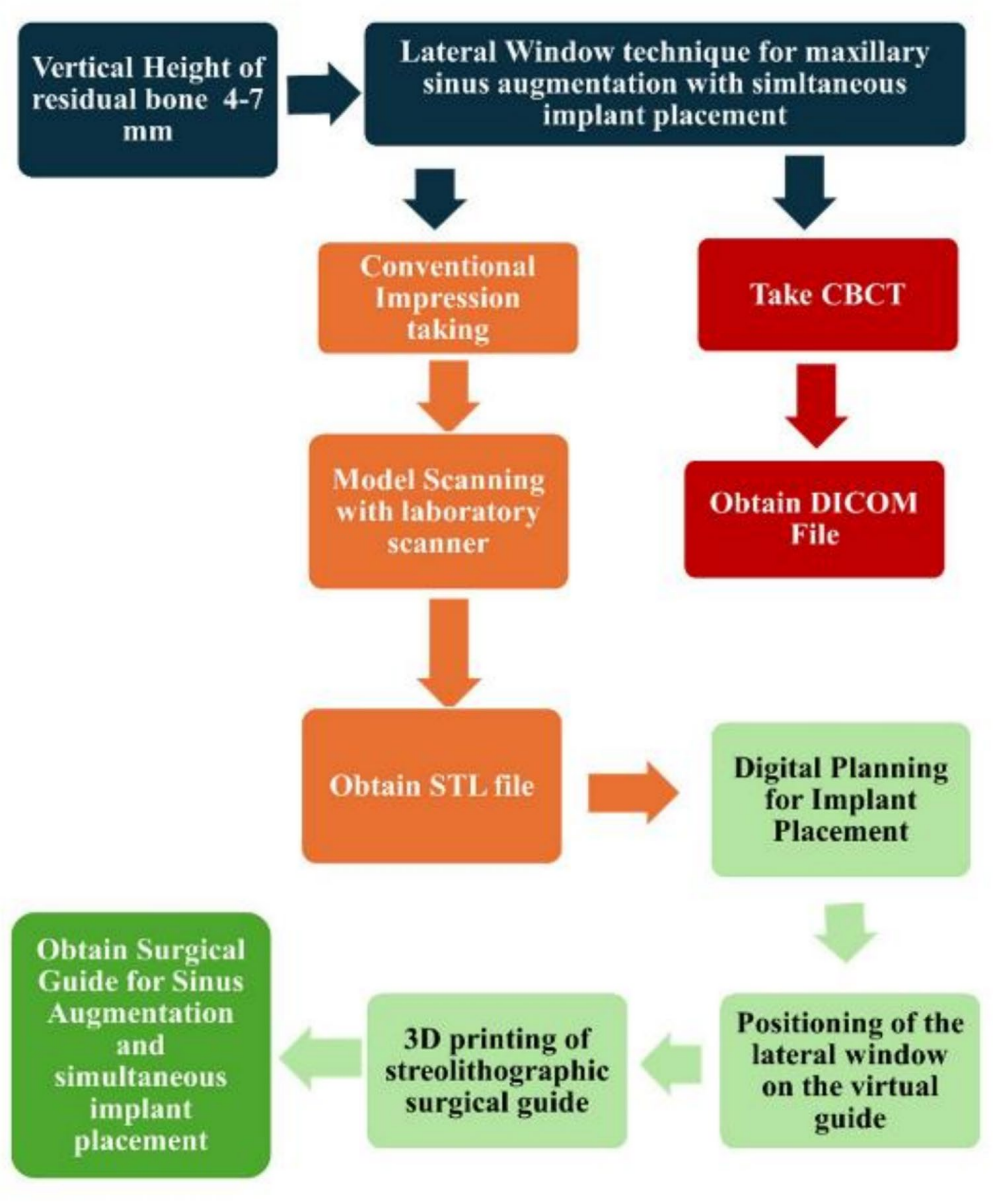
Workflow for study group

The workflow [Fig. 2] includes obtaining the DICOM files from the CBCT, scanning the diagnostic models to obtain the STL files to virtually plan the stereolithographic surgical guide and the virtual position of the lateral window. Then the stereolithographic surgical guide is printed using a 3D printer and used during surgery.

### Preoperative scanning

Preoperative cone beam computed tomographic radiographs using the Scanora three-dimensional imaging system using CMOS flat panel detector with isotropic voxel size 133 μm, the x-ray tube used to scan the patients possess a current intensity 10 mA, 90 kVp and a focal spot size 0.5 mm. The scanning time was 14 s of pulsed exposure resulting in an effective exposure time of 3.2 s to scan FOV (field of view) of 14 cm height × 16.5 cm width. FOV adjustment was guided by three laser light beams to centralize the area of interest within the scanning field. The primary reconstruction time for DICOM data set was two minutes. Then, the raw DICOM data set images were imported to the On-Demand software (On Demand—Cybermed, Seoul, Korea) for secondary reconstruction and image analysis.

Each patient was evaluated for bone quantity and quality, mesio-distal distance, bucco-lingual dimension of the potential implant insertion site, as well as its relation to the nasal cavity, maxillary sinus and the evaluation of major carious lesions in the remainder of the dentition and the detection of the remaining roots or any suspected pathological lesions.

Cone beam computed tomographic evaluation was performed to allow for a more comprehensive overall view and better interpretation of the anatomic structures. As well as the patients who revealed neighboring remaining roots or carious lesions were planned for treatment.

### Stereolithographic surgical guide fabrication

For the **study group** the cast was scanned using Scanora three-dimensional imaging system and exported as a standard tessellation language (STL) file, while the CBCT image was saved as Digital Imaging and Communications in Medicine (DICOM) data.

The Stereolithographic Surgical Guide was fabricated by superimposition of the STL file on the CBCT through the In2 guide module of Ondemand 3D software. The proper position of the lateral window will be planned and the same would be done for the implant(s) position(s). After proper adjustment, the adequate position of the implant was planned with the creation of metal sleeve. In cases where there were adjacent teeth on the mesial or the distal side, at least two adjacent teeth were included in the surgical guide.

In addition, the appropriate location of the lateral window was planned. Adjustment of its mesiodistal position was made with consideration on the positions of the third molar, sinus septa, and adjacent teeth or implants. The mesial and the distal boundaries of the window were set at least 1.5 mm away from the adjacent teeth or implants and would be 3 mm away from the inferior border of the maxillary sinus**.**

**Fig. 3:**
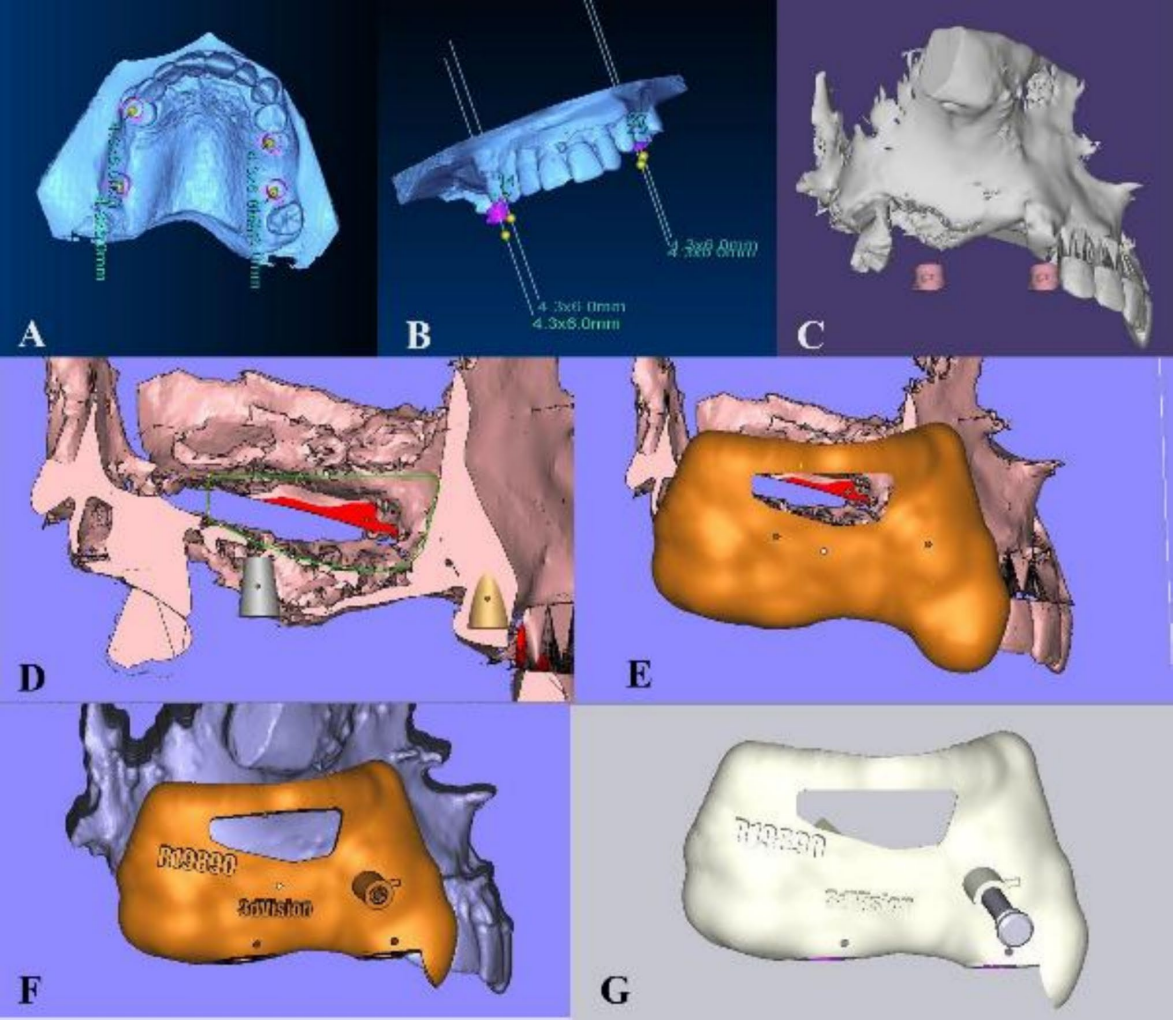
**A**: Sleeve position on virtual cast. **B**: Sleeve angulation on virtual cast. **C**: Sleeve position on the 3D reconstructed model buccal view. **D**: Planned implant position in relation to maxillary sinus on virtual cast buccal view. **E**: Lateral window position planning in the planned stereolithographic surgical guide. **F**: Planned stereolithographic surgical guide with the anchor pin position. **G**: Planned stereolithographic surgical guide

When the lateral window location is set, it is then removed from the printed guide as a rectangular shape to imitate the shape of the lateral window opening. The completed design of the surgical guide will then be exported as an STL file. The virtual surgical guide was imported and printed using 3D printer (Envision Tech Inc., Dearborn, Michigan, U. S). [Fig. 3].

**Fig. 4:**
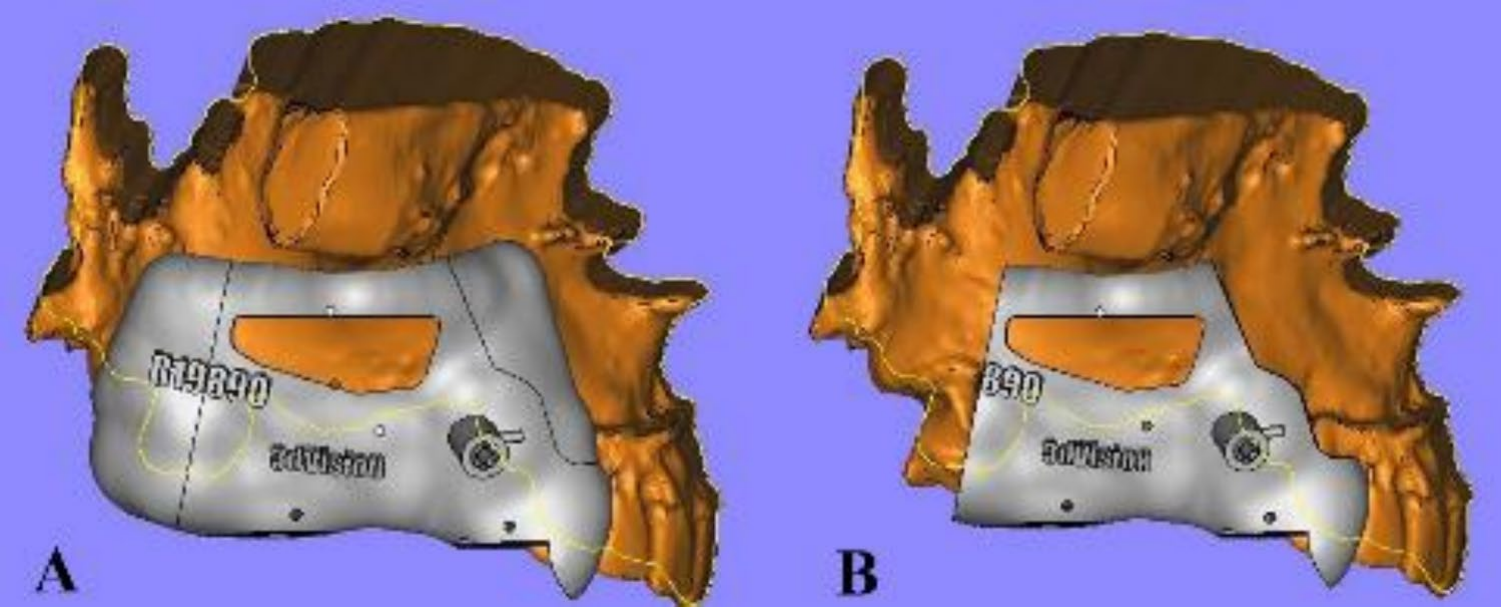
**A**: Planned stereolithographic surgical guide with the modification requested. **B**: Planned stereolithographic surgical guide after modification from buccal view

Modifications were made to the design after the first two cases as extensions of the stereolithographic guide were excessive and required a larger flap and were reduced during surgery to allow for proper seating, therefore consuming more time [Fig. 4].

The surgical guide was sterilized using gamma irradiation process prior to the surgery**.** The stereolithographic surgical guide design would be tooth-bone supported and would guide the four sides of the lateral window osteotomy.

### For both groups

#### Preoperative care:

All subjects were asked to rinse with an antiseptic chlorohexidine mouthwash (Hexitol Mouthwash, ADCO, Egypt) for one minute prior to the surgical procedure.

### Surgical procedure

Preoperative photographs were taken prior to the surgical procedure. Local anesthesia (Articane 4%) (Artinibsa 4%, Inibsa, Spain.) was administered using infiltration technique buccal and lingual.

A Crestal incision and a releasing vertical incision mesial to the adjacent tooth and distal to the surgical field was performed – producing a full thickness trapezoidal flap, to expose the alveolar ridge and lateral wall of the maxillary sinus.

#### For the study group:

**Fig. 5:**
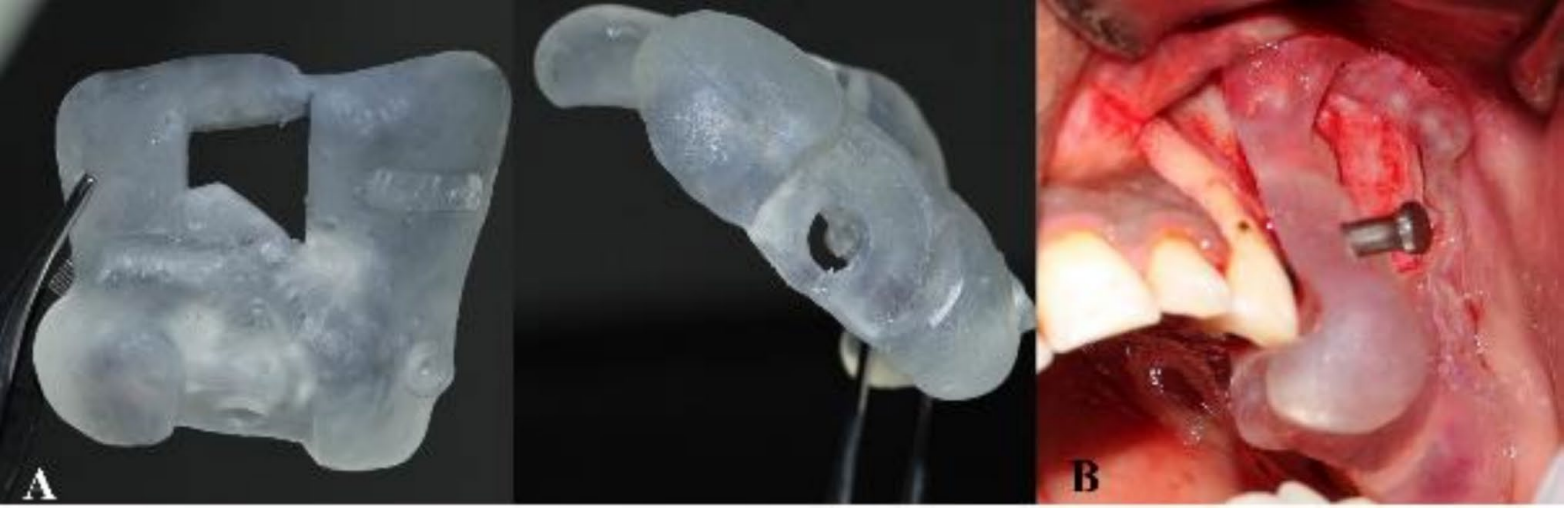
**A**: Stereolithographic Surgical Guide. **B**: Stereolithographic Surgical Guide anchored in position

The prefabricated guide was adapted firmly to the surgical site using stabilizing pins [Fig. 5]. Using a round diamond bur used on a 45° contra-angle handpiece the outlines of the lateral window osteotomy were performed guided by the surgical guide.

**Fig. 6:**
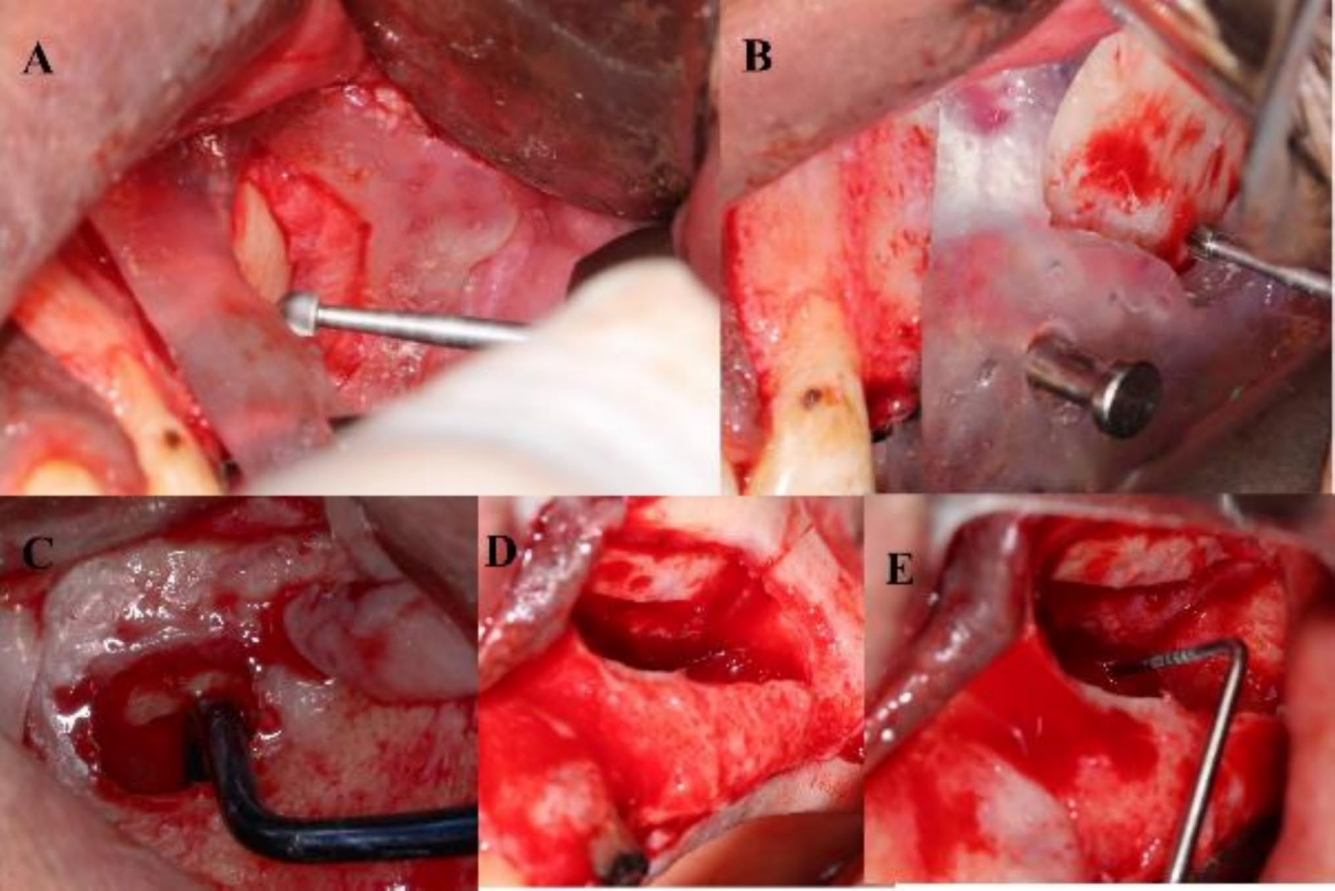
**A**: Round diamond bur performing the lateral window osteotomy through the Surgical Guide. **B**: Boundaries of the lateral window osteotomy through the Surgical Guide. **C**: Open sinus kit instruments used to elevate the Schneiderian membrane. **D**: Elevation of the Schneiderian membrane with the buccal wall bone as the new inferior border of the maxillary sinus. **E**: A periodontal probe used to inspect the palatal bone to make sure the Schneiderian membrane is elevated completely

Once the lateral window was performed, the surgical guide was removed, and the sinus membrane was carefully elevated along the inferior, lateral and medial walls to avoid iatrogenic perforations using sinus elevation kit. A periodontal probe is then used to inspect the palatal bone to make sure the Schneiderian membrane is elevated completely [Fig. 6].

**Fig. 7:**
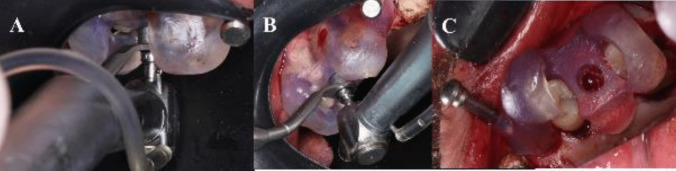
**A**: Drilling implant osteotomy through the guide. **B**: Implants drilling through the guide. **C**: Osteotomy seen through the guide

The surgical guide was then reinstalled, and pilot drills were used to mark the position of the implant(s). Subsequent drilling of implant osteotomies was then performed [Fig. 7].

**Fig. 8:**
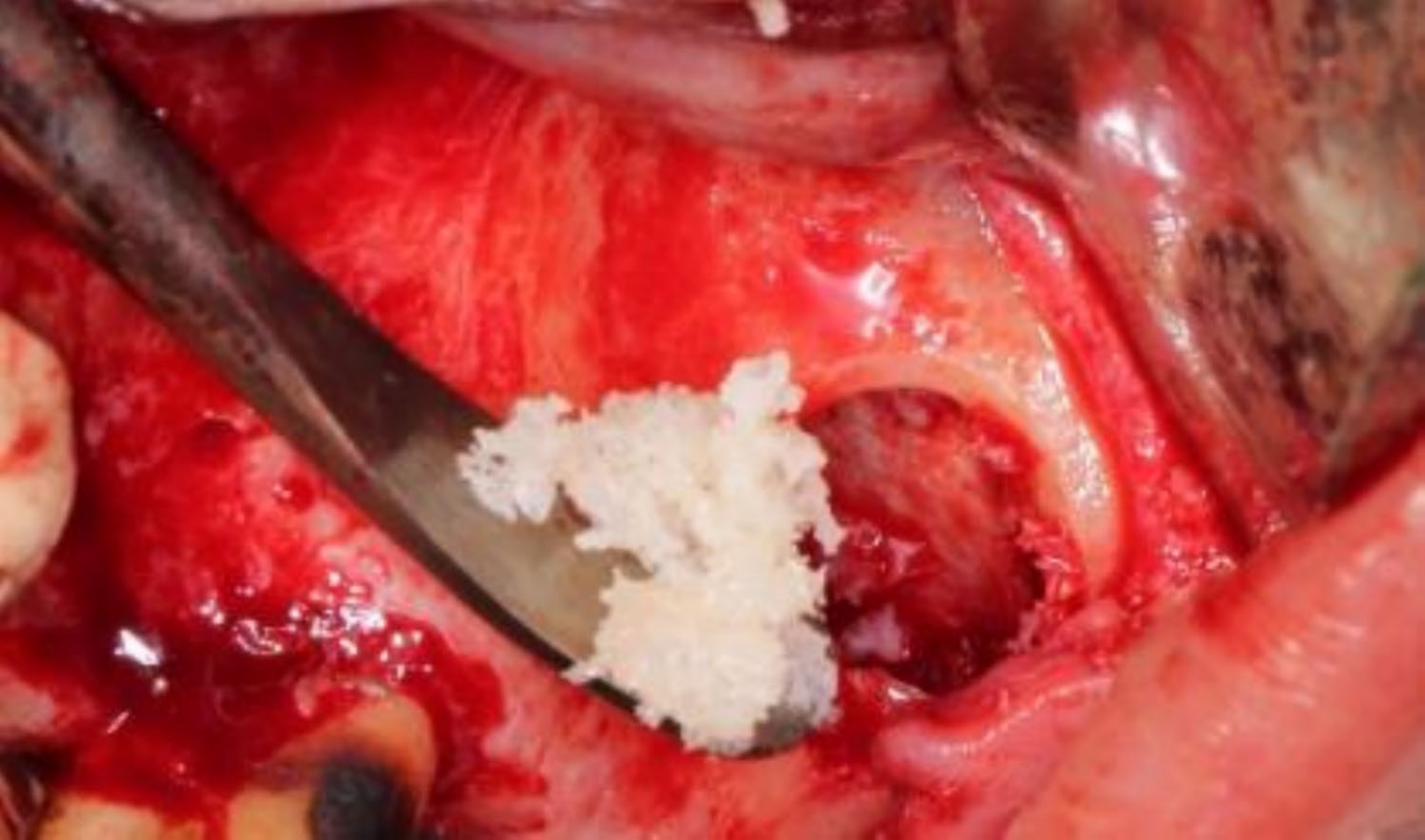
Placement of bone graft prior to implant placement palatally and medially

The surgical Guide was then removed and the allograft bone material (Maxxeus, Center for Tissue, Innovation and Research Manufacturing and Distribution Center 2900, College Dr. Kettering, OH 45420) was placed palatally and medially between the elevated Schneiderian membrane and the alveolar crest [Fig. 8].

**Fig. 9:**
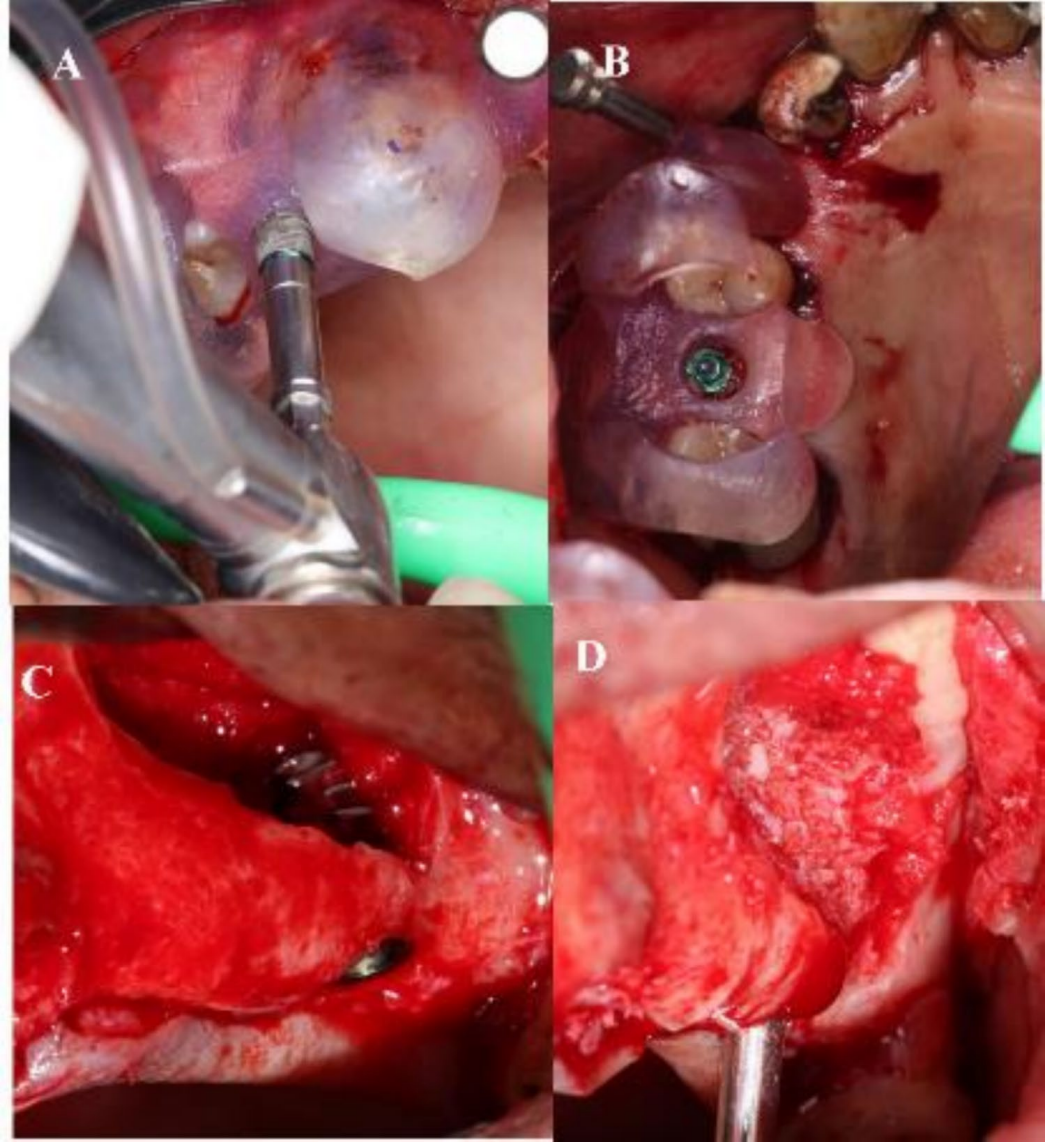
**A**: Implants placed through the guide. Figure **B**: Implant position through the guide. **C**: Implant in position in relation to the elevated maxillary sinus. Figure **D**: Bone graft in position

The implant(s) (NUVO™ Internal FIT™, Straumann Holding AG, Straumann North American Headquarters Straumann USA, LLC 60 Minuteman Road | Andover, MA 01810) were then placed., and then the allograft bone material was packed in the window to completely fill the space between the elevated Schneiderian membrane and alveolar crest. A resorbable collagen membrane was then placed buccally to seal the window, covering the allograft bone material [Fig. 9]. That was followed by the suturing of the flap using vicryl 3–0 suture material (3–0 Vicryl Suture, Assut Medical, Switzerland).

#### For the control group:

**Fig. 10:**
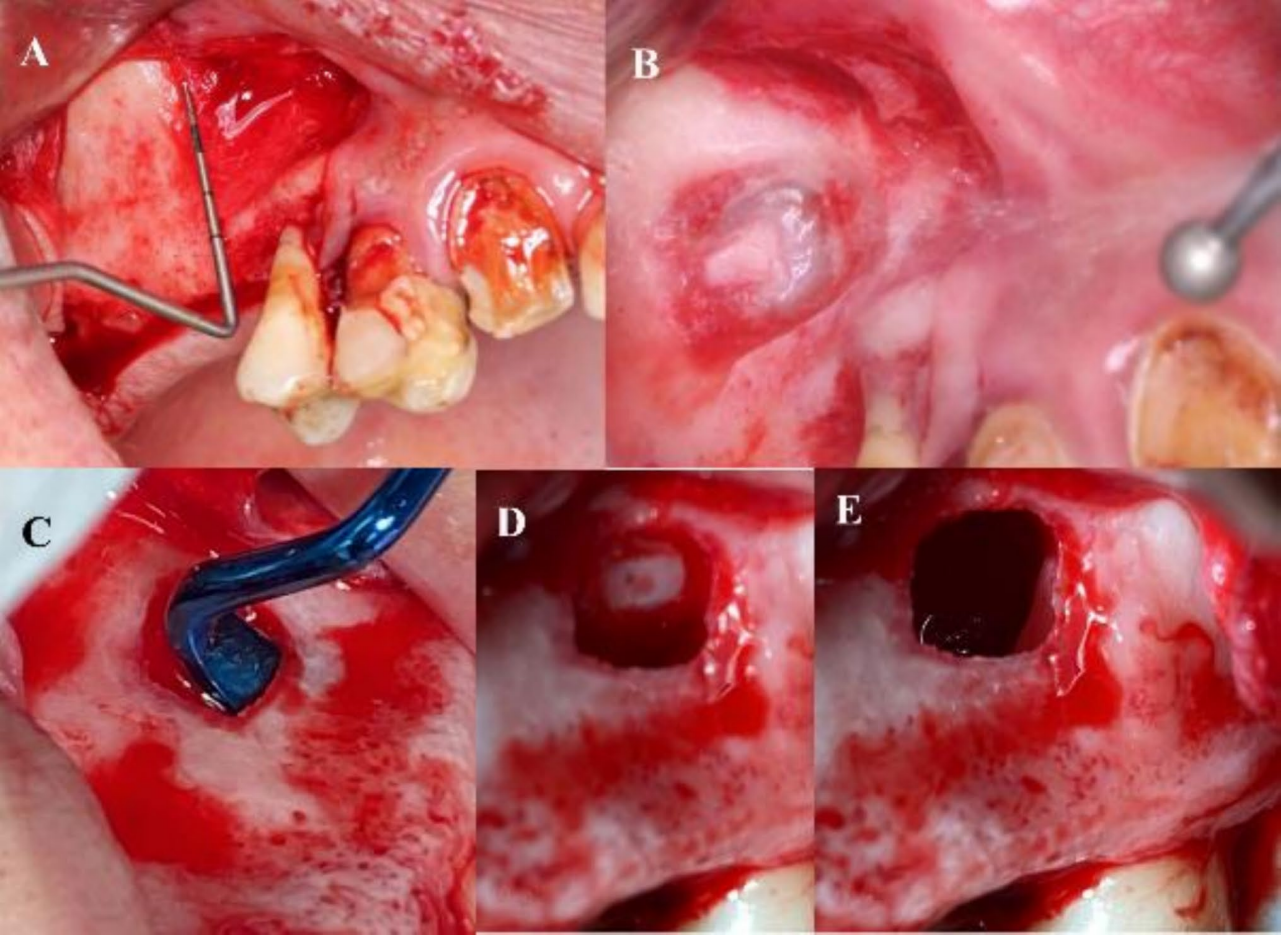
**A**: Measurements of the lateral window boundaries using periodontal probe. **B**: Schneiderian membrane shadow in osteotomy. **C**: Schneiderian membrane elevation. **D**: Schneiderian membrane elevated with trap door seen as the roof of the osteotomy. E: Schneiderian membrane after the completion of lateral window osteotomy

After the flap reflection using a periodontal probe the margins of the window are marked**.** Using a round diamond bur used on a 45° contra-angle handpiece the outlines of the lateral window osteotomy were performed free hand. Then the sinus membrane was carefully elevated along the inferior, lateral and medial walls to avoid iatrogenic perforations. Implant osteotomies were made according to manufacturer’s instructions and the allograft bone material (Maxxeus, Center for Tissue, Innovation and Research Manufacturing and Distribution Center 2900 College Dr. Kettering, OH 45420) was placed palatally and laterally between the elevated Schneiderian membrane and the alveolar crest [Fig. 10].

**Fig. 11:**
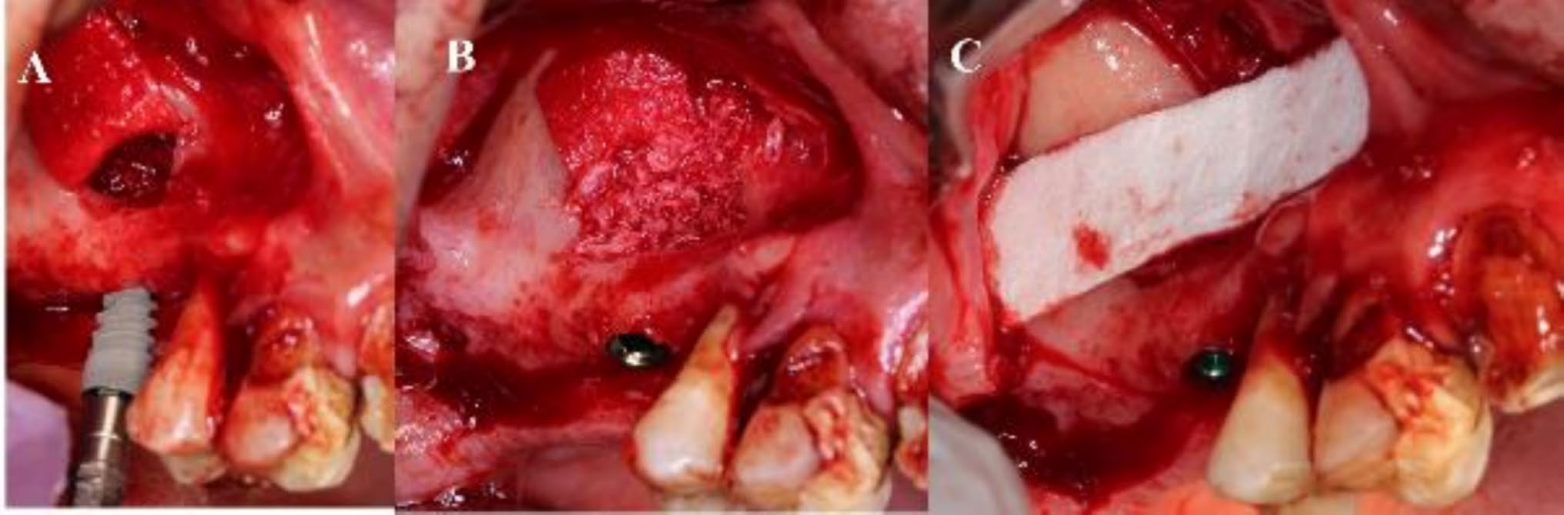
**A**: Implant placement. **B**: Bone graft packed in osteotomy. **C**: collagen membrane placement over the lateral window osteotomy

The implant(s) (NUVO™ Internal FIT™ Straumann Holding AG, Straumann North American Headquarters Straumann USA, LLC 60 Minuteman Road | Andover, MA 01810) were then placed, and then the allograft bone material was packed in the window to complete fill the space between the elevated Schneiderian membrane and alveolar crest. A resorbable collagen membrane was then placed buccally to seal the window, covering the allograft bone material. That was followed by the suturing of the flap using vicryl 3–0 suture material [Fig. 11].

#### Post-operative care:

After placement of the implant and delivery of the temporary restorations (partial dentures), antibiotics were prescribed Amoxycillin and clavulanate potassium 1 gm oral tablet (Augmentin 1 g (oral), gsk GlaxoSmithKline, Egypt.) twice daily for 5 days postoperatively (or clindamycin 300 mg (Dalacin—C 300 mg (oral), Phizer, Egypt.) in patients who are allergic to penicillin 3 times daily for 5 days postoperatively). Postoperative analgesia (non-steroidal anti-inflammatory drugs (Cataflam, Novartis Pharmaceuticals Corporation, Egypt.)) was prescribed for 3 days, and then whenever it is needed for pain relief. The patient was also given decongestant nasal drops (Otrovin Nasal Drops, Novartis, Egypt). The patient was instructed to rinse their mouth with antiseptic mouthwash (Chlorohexidine) three times a day starting from the second day postoperatively and continued for two successive weeks.

CBCT of the implants site were accomplished immediately postoperatively to verify the position of the implant and the extent of sinus elevation and for comparison between the preoperative and postoperative alveolar bone height and bone density.

#### Follow up phase:

All subjects were evaluated based on the following timeline**.**

- **One-week recall**, all subjects were recalled checking for the presence of infection, to evaluate the oral hygiene of the subjects, and to remove the sutures.
- **One month postoperatively**, to evaluate the overall healing process, to prevent any complications that could occur and to control the subjects overall oral hygiene.
- **6 months postoperatively**, for stage 2 uncovering surgery, placement of healing abutments. Eventually steps for construction of the final prosthesis were accomplished at this stage.

### Method of evaluation:

#### Clinical evaluation:

1. **Presence or absence of intra or postoperative complications**

The presence or absence of intraoperative or postoperative complications were recorded in the patient’s file.

Those complications included in the study were:

- **Schneiderian membrane perforation occurrence**
- **intraoperative bleeding**
- **epistaxis**
- **postoperative sinusitis**
- **periimplantitis**

2. **Postoperative pain**

**Fig. 12:**
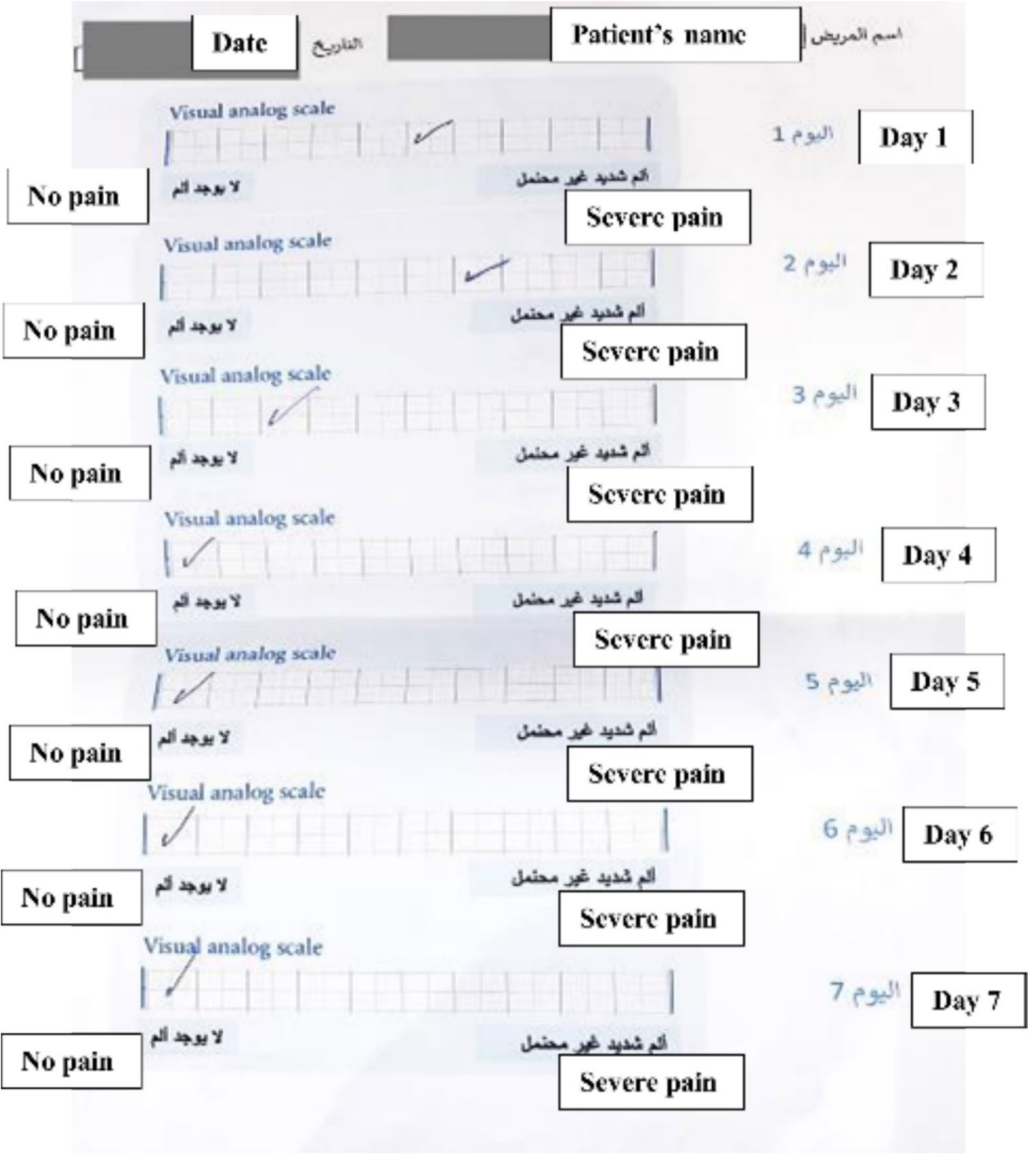
Pain VAS filled by one of the patients. (text boxes represent English translation of the original VAS that was designed in Arabic (mother tongue of participants))

Postoperative pain was recorded by asking the patient to fill in a Visual Analogue scale [Fig. 12] for the ten-day period following the surgical procedure. This was then recorded in the patient’s file by the primary investigator of this study.

The visual analog scale (VAS) was used to assess postoperative pain severity. The VAS tool was a 10- cm ruler displaying 0 (no pain) on one end and 10 (intense pain) on the other. A VAS score lower than three indicated mild pain, a score of 3–7 indicated moderate pain, and a score higher than seven indicated severe pain, according to the literature [28].

3. **Postoperative edema**

**Fig. 13:**
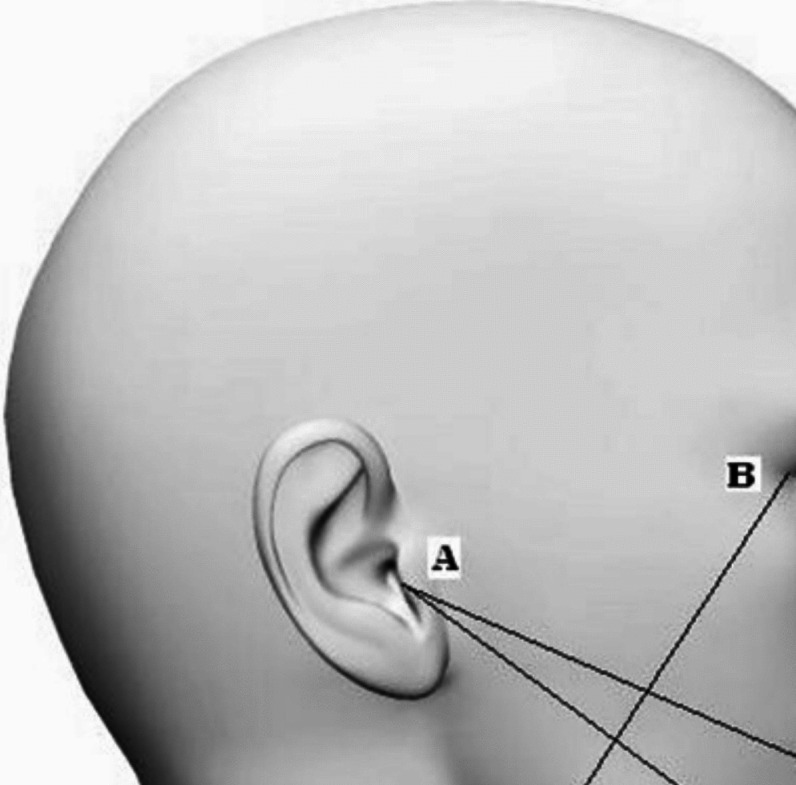
Five facial points for edema analysis: The most posterior point on the tragus **A**, the lateral canthus of the eye (B), the most lateral point on the mouth corner **C**, the soft tissue pogonium, which is the most prominent point on the chin **D**, and the most inferior point on the mandibular angle (E) [29]

**Fig. 14:**
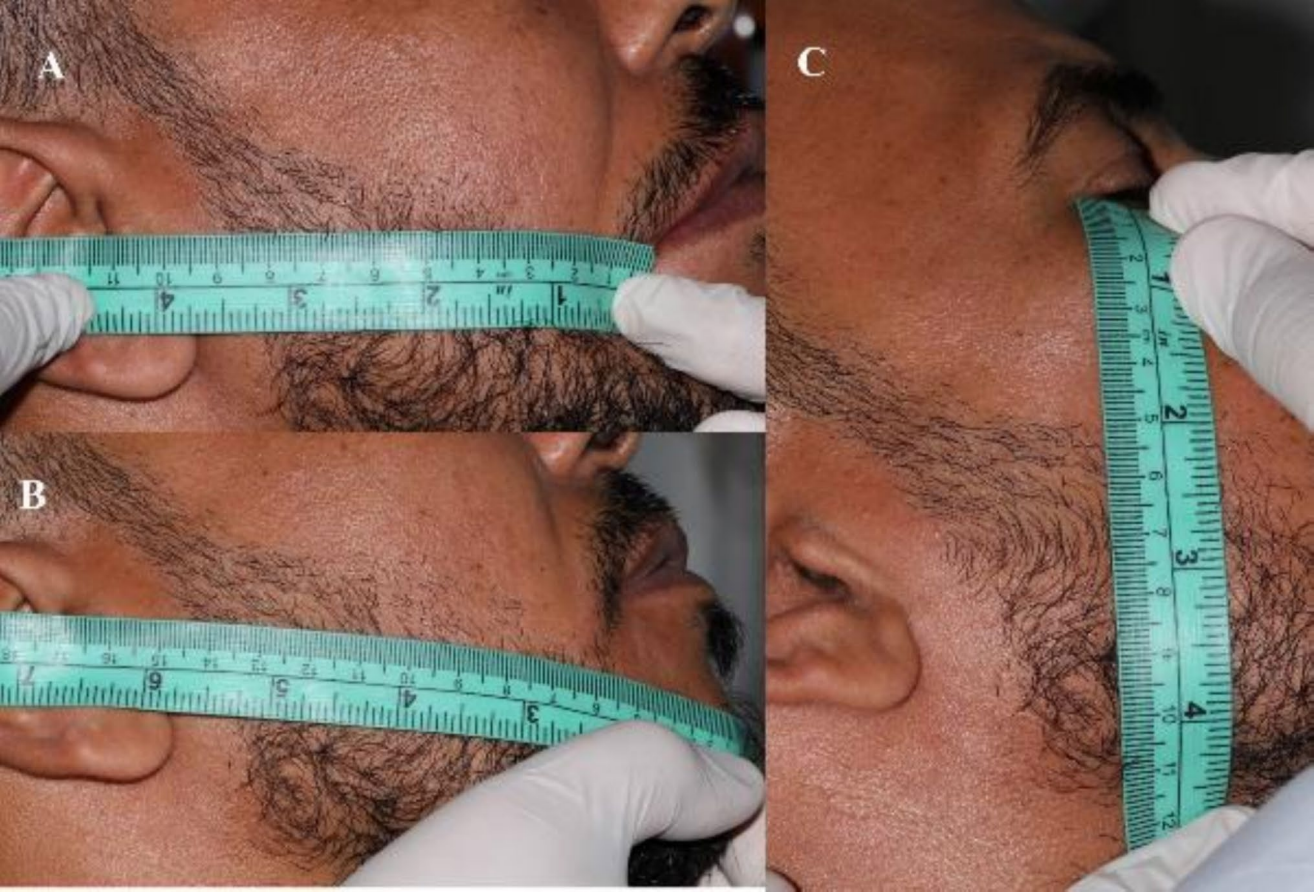
**A**: The measures from point **A** (The most posterior point on the tragus) to point **C** (the most lateral point on the mouth corner). **B**: The measures from point **A** (The most posterior point on the tragus) to point **D** (, which is the most prominent point on the chin) **C**: The measures from point **B** (lateral canthus of the eye) to point E (the most inferior point on the mandibular angle)

To attain the measurement of the edema scale of the patients in both groups the facial measurements had to first be recorded before surgery. With the patient seated upright and the mandible in the physiological rest position, measurements of the patient's face were taken with a measuring tape. The study employed five facial points for analysis as is shown in [Fig. 13, 14]. The most posterior point on the tragus (A), the lateral canthus of the eye (B), the most lateral point on the mouth corner (C), the soft tissue pogonium, which is the most prominent point on the chin (D), and the most inferior point on the mandibular angle (E)**.** The measures for these three lines—A to C, B to E, and A to D—were taken three times, with an average being determined [29].

On the third day postoperative, the patient’s same facial measurements that were taken before surgery will be repeated in the same way to record the scale of edema. The edema was calculated after subtracting the pre-operative measurement from the 3-day postoperative measurement [29].

Another evaluation for the edema was done using the edema Visual Analogue Scale where the edema ranks “None” (no inflammation), “Mild” (intraoral swelling confined to the surgical field), “Moderate” (extraoral swelling in the surgical zone), “Severe” (extraoral swelling spreading beyond the surgical zone).

4. **Implant stability**

Implant stability was measured using the Osstell device immediately following implant placement and 6 months postoperatively**.**

### Radiographic evaluation:

#### Automated voxel superimposition method of CBCT scans:

To ensure standardization and reproducibility of the CBCT cross sectional images used in this study, superimposition of DICOM sets of each patient using Fusion module of Ondemand 3D App software was done. This 3D superimposition technique allows for sub-voxel accuracy and highly robust registration. Importing the low DICOM sets to the Fusion module of Ondemand 3D software Both files are loaded in the Fusion module at the time, first manual registration was done by approximation of the secondary scan to the primary one in axial, sagittal and coronal cuts, then automatic registration was done by the software. Cross sectional slices were obtained with the long axis of the implant in the superimposed axial, sagittal and coronal cuts, with slice thickness of 1.5 mm. these slices had the same coordination in both sets thus linear measurements could be made at the exact same sites in the primary and secondary scans simultaneously to measure the buccolingual thickness of the bone at the selected.

5. **Vertical bone height gained**

**Fig. 15:**
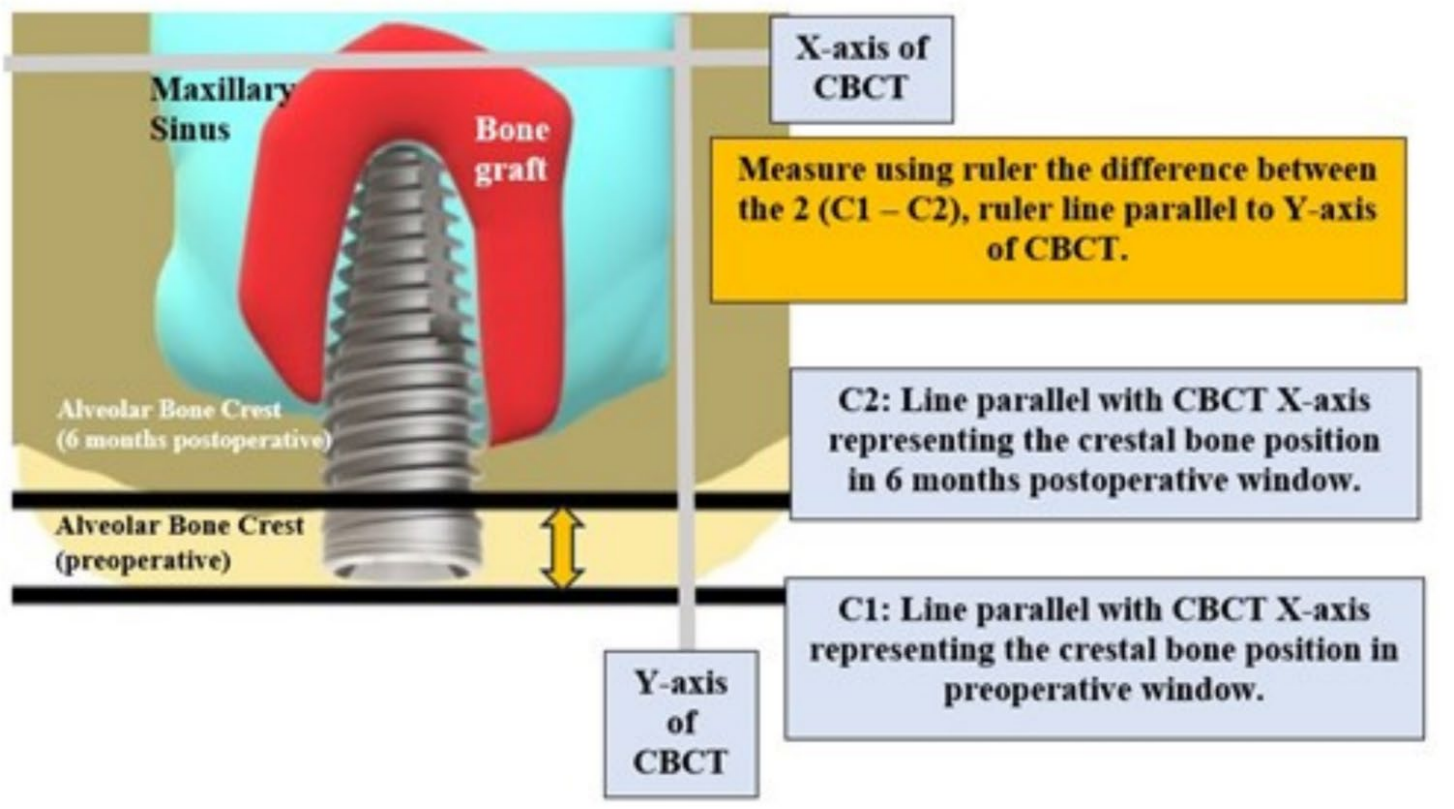
Method for measurement of vertical bone gain

**Fig. 16:**
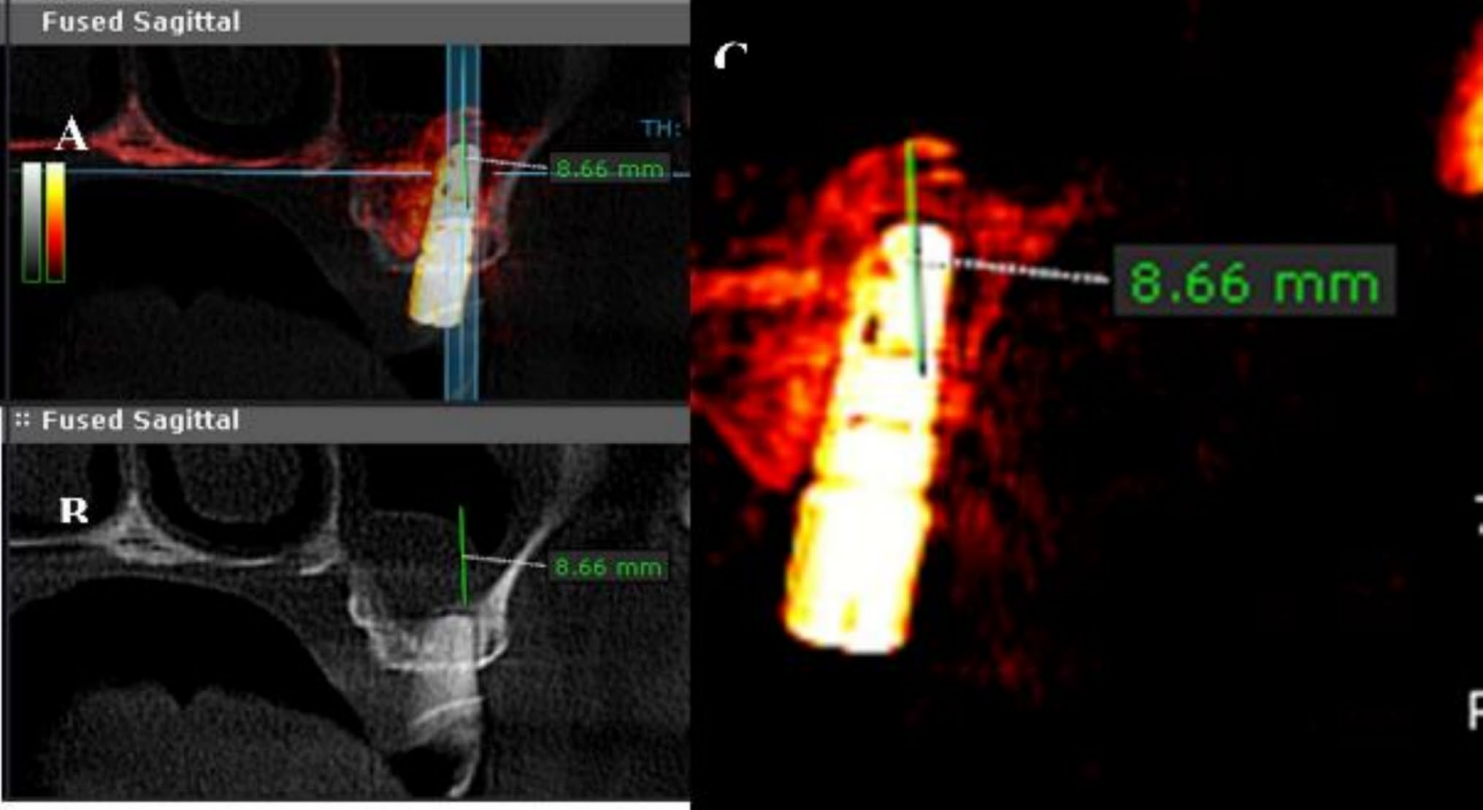
**A**: Fused Sagittal window. **B**: The preoperative window of fusion module. **C**: The 6 months postoperative window of fusion module

Using the fusion module of CBCT on the Fused Sagittal view a window showing the 6 months CBCT (in red) superimposed on the preoperative CBCT (grey) a point was made at the highest point of the bone graft using the ruler tool and extended to a point on the inferior border of the maxillary sinus, the ruler line measured must be parallel to Y-axis of CBCT as shown in [Fig. 15, 16].

### Statistical analysis

Numerical data were explored for normality by checking the distribution of data and using tests of normality (Kolmogorov–Smirnov and Shapiro–Wilk tests). Age, torque, and implant stability data showed normal (parametric) distribution while edema scale, satisfaction, pain scores as well as bone changes data showed non-parametric distribution. Data were presented as mean, standard deviation (SD), median and range values. For parametric data, Student’s t-test was used to compare between mean ages in the two groups. Repeated measures ANOVA test was used to compare between the two groups as well as to study the changes by time within each group. Bonferroni’s post-hoc test was used for pair-wise comparisons when the ANOVA test is significant. For non-parametric data, the Mann–Whitney U test was used to compare between the two groups. Wilcoxon-signed rank test was used to study the changes by time within each group. Friedman’s test was used to study the changes in pain scores within each group. Dunn’s test was used for pair-wise comparisons when Friedman’s test is significant. Qualitative data were presented as frequencies and percentages. Chi-square and Fisher’s Exact tests were used to compare between the two groups regarding qualitative data. The significance level was set at P ≤ 0.05. Statistical analysis was performed with IBM SPSS Statistics for Windows, Version 23.0. Armonk, NY: IBM Corp.

## Results

### Baseline characteristics

Study group showed statistically significantly higher prevalence of females than control group. There was no statistically significant difference between mean age values in the two groups. There was also no statistically significant difference between mean torque in the two groups [Table 1].

**Table 1:** Descriptive statistics and results of Fisher’s Exact test and Student’s t-test for comparisons between base line characteristics in the two groups

Base line characteristics	Study (n = 15)	Control (n = 15)	P-value
Gender [n, (%)]			
Male	1 (6.7%)	9 (60%)	0.001*
Female	14 (93.3%)	6 (40%)	
Age in years [Mean, SD]	42.1 (7.5)	40.3 (11.5)	0.609
Torque in N/cm [Mean, SD]	26.5 (15.6)	34.7 (18.1)	0.188

^*^: Significant at P ≤ 0.05

### Implant success

The 12-month implant survival rate was 100% (15/15) in the guided group versus 93.3% (14/15) in the freehand group (p = 1). There was no statistically significant difference between implant success in the two groups [Table 2].

**Table 2:** Descriptive statistics and results of Fisher’s Exact test for comparisons between implant success in the two groups

	Study (n = 15)	Control (n = 15)	P-value
Implant success [n, (%)]			
Success	15 (100%)	14 (93.3%)	1
Failure	0 (0%)	1 (6.7%)	

^*^: Significant at P ≤ 0.05

### Complications

There was no statistically significant difference between prevalence of Schneiderian membrane perforation and post-operative sinusitis in the two groups. None of the cases in the two groups showed intra-operative bleeding, epistaxis or peri-implantitis [Table 3].

**Table 3:** Descriptive statistics and results of Fisher’s Exact test for comparisons between presence of complications in the two groups

Complications [n, (%)]	Study (n = 15)	Control (n = 15)	P-value
Schneiderian membrane perforation			
Present	1 (6.7%)	2 (13.3%)	1
Absent	14 (93.3%)	13 (86.7%)	
Post-operative sinusitis			
Present	2 (13.3%)	2 (13.3%)	1
Absent	13 (86.7%)	13 (86.7%)	

^*^: Significant at P ≤ 0.05

#### Pain (VAS scores)

### Comparison between the two groups

After one day, there was no statistically significant difference between the two groups (*P*-value = 0.114, Effect size = 0.576). After two days, the study group showed a statistically significantly lower pain score than control group (*P*-value = 0.0121, Effect size = 1). After three, four, five, six, seven, eight, nine as well as ten days, there was no statistically significant difference between the two groups (*P*-value = 0.236, Effect size = 0.428), (*P*-value = 0.146, Effect size = 0.537), (*P*-value = 0.170, Effect size = 0.505), (*P*-value = 0.340, Effect size = 0.346), (*P*-value = 0.188, Effect size = 0.474), (*P*-value = 0.826, Effect size = 0.078), (*P*-value = 0.780, Effect size = 0.085) and (*P*-value = 0.302, Effect size = 0.114), respectively.

### Changes within each group

**Fig. 17:**
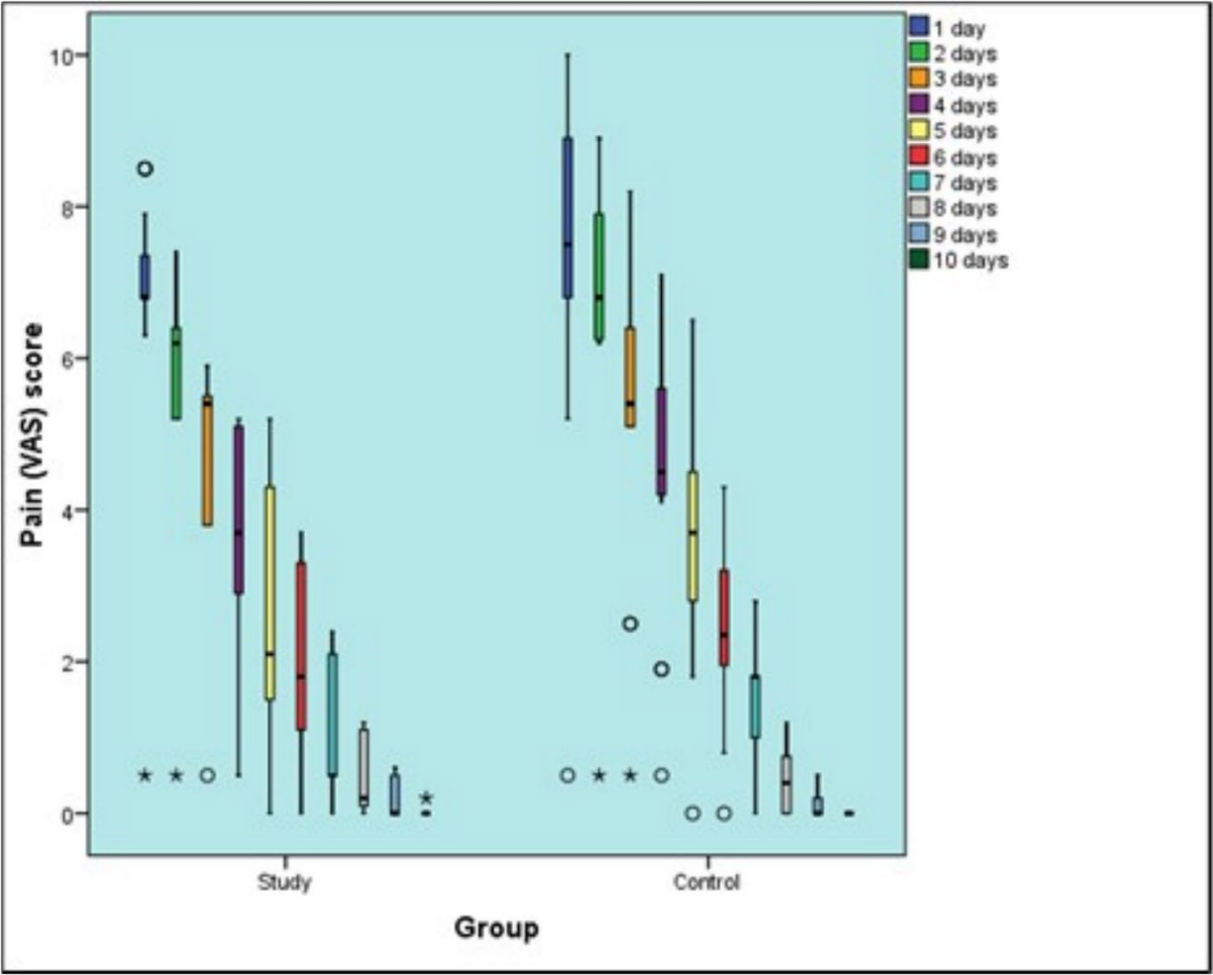
Box plot representing median and range values for pain scores in the two groups (Circles and stars represent outliers)

In the study group, there was a statistically significant change in pain scores by time (*P*-value < 0.001, Effect size = 0.979). Pair-wise comparisons between time periods revealed that there was no statistically significant change in pain scores after two days. There was a statistically significant decrease in pain scores from two to three, three to four as well as four to five days followed by a non-statistically significant change in pain scores from five to six days. From six to seven as well as seven to eight days, there was a statistically significant decrease in pain scores. Fron eight to nine as well as nine to 10 days, there was no statistically significant change in pain scores. As regards control group, there was a statistically significant change in pain scores by time (*P*-value < 0.001, Effect size = 0.977). Pair-wise comparisons between time periods revealed that there was no statistically significant change in pain scores after two days followed by a statistically significant decrease in pain scores from two to three, three to four, four to five, five to six, six to seven as well as seven to eight days. There was no statistically significant change in pain scores from eight to nine as well as nine to 10 days [Table 4] [Fig. 17].

**Table 4:** Descriptive statistics and results of Mann–Whitney U test for comparison between pain (VAS) scores in the two groups and Friedman’s test for the changes within each group

	Study (n = 15)		Control (n = 15)		P-value	Effect size (d)
	Median (Range)	Mean (SD)	Median (Range)	Mean (SD)		
1 day	6.8 (0.5, 8.5) ^A^	6.76 (1.89)	7.5 (0.5, 10) ^A^	7.34 (2.33)	0.114	0.576
2 days	6.2 (0.5, 7.4) ^A^	5.75 (1.67)	6.8 (0.5, 8.9) ^A^	6.77 (1.94)	0.012*	1
3 days	5.4 (0.5, 5.9) ^B^	4.64 (1.44)	5.4 (0.5, 8.2) ^B^	5.34 (2.05)	0.236	0.428
4 days	3.7 (0.5, 5.2) ^C^	3.73 (1.31)	4.5 (0.5, 7.1) ^C^	4.51 (1.84)	0.146	0.537
5 days	2.1 (0, 5.2) ^D^	2.66 (1.54)	3.7 (0, 6.5) ^D^	3.68 (1.7)	0.170	0.505
6 days	1.8 (0, 3.7) ^D^	2.07 (1.21)	2.35 (0, 4.3) ^E^	2.44 (1.24)	0.340	0.346
7 days	0.5 (0, 2.4) ^E^	1.07 (0.84)	1.8 (0, 2.8) ^F^	1.56 (0.82)	0.188	0.474
8 days	0.2 (0, 1.2) ^F^	0.5 (0.49)	0.4 (0, 1.2) ^G^	0.47 (0.43)	0.826	0.078
9 days	0 (0, 0.6) ^F^	0.17 (0.25)	0 (0, 0.5) ^G^	0.11 (0.17)	0.780	0.085
10 days	0 (0, 0.2) ^F^	0.01 (0.05)	0 (0, 0) ^G^	0 (0)	0.302	0.114
P-value	< 0.001*		< 0.001*			
Effect size (d)	0.979		0.977			

^*^: Significant at P ≤ 0.05, Different superscripts within the same column indicate statistically significant change by time

#### Edema scale

**Fig. 18:**
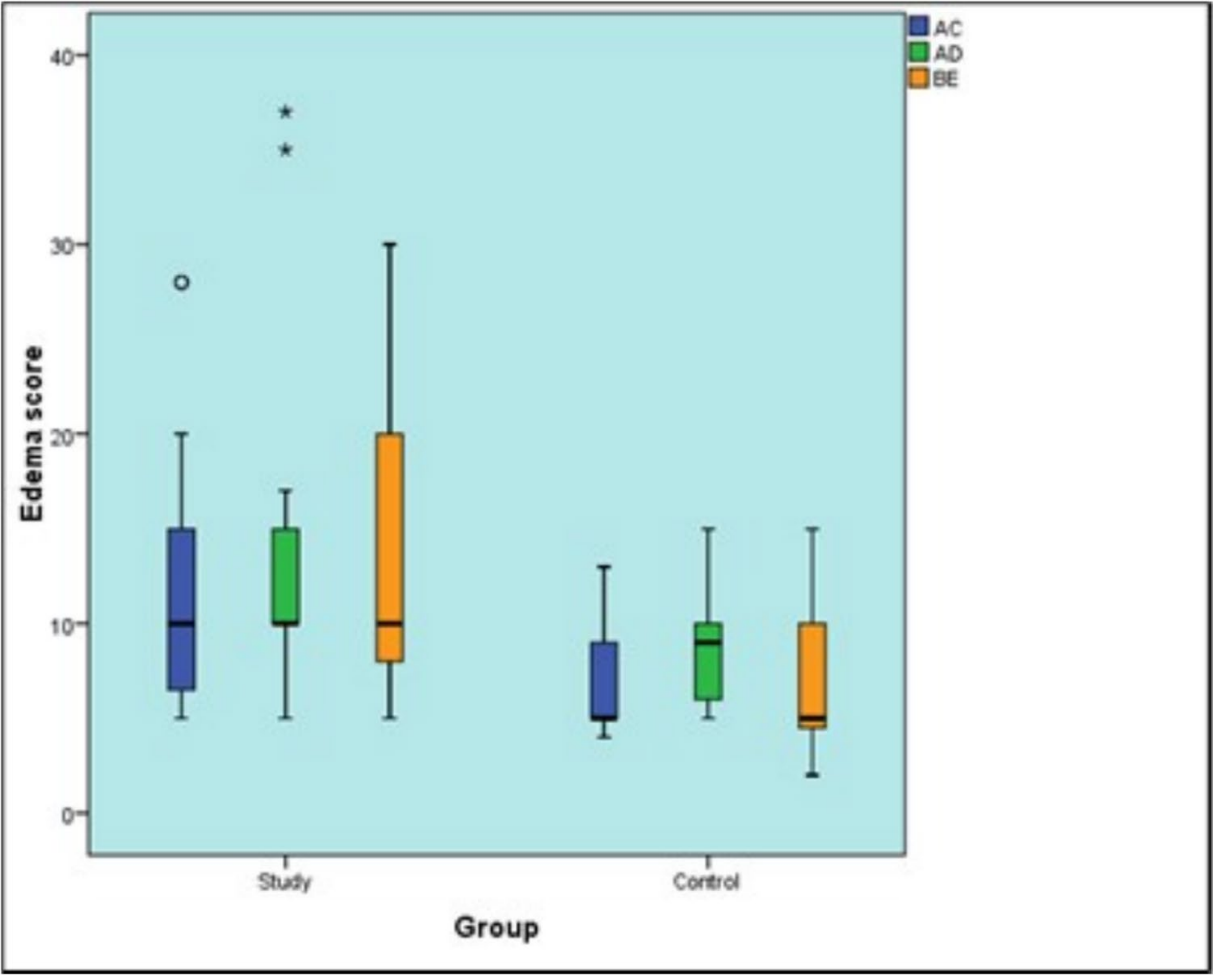
Box plot representing median and range values for edema scale scores in the two groups (Stars and circle represent outliers)

As regards AC, AD as well as BE, the study group showed statistically higher scores than control group (*P*-value = 0.003, Effect size = 1.223), (*P*-value = 0.030, Effect size = 0.812) and (*P*-value = 0.005, Effect size = 1.156), respectively [Table 5] [Fig. 18].

**Table 5:** Descriptive statistics and results of Mann–Whitney U test for comparison between edema scale in the two groups

Edema scale	Study (n = 15)		Control (n = 15)		P-value	Effect size (d)
	Median (Range)	Mean (SD)	Median (Range)	Mean (SD)		
AC	10 (5, 28)	12.4 (7.63)	5 (4, 13)	6.5 (2.76)	0.003*	1.223
AD	10 (5, 37)	14.6 (9.39)	9 (5, 15)	9.06 (3.53)	0.030*	0.812
BE	10 (5, 30)	14.27 (8.13)	5 (2, 15)	6.81 (3.51)	0.005*	1.156

^*^: Significant at P ≤ 0.05

#### Severity of edema

**Fig. 19:**
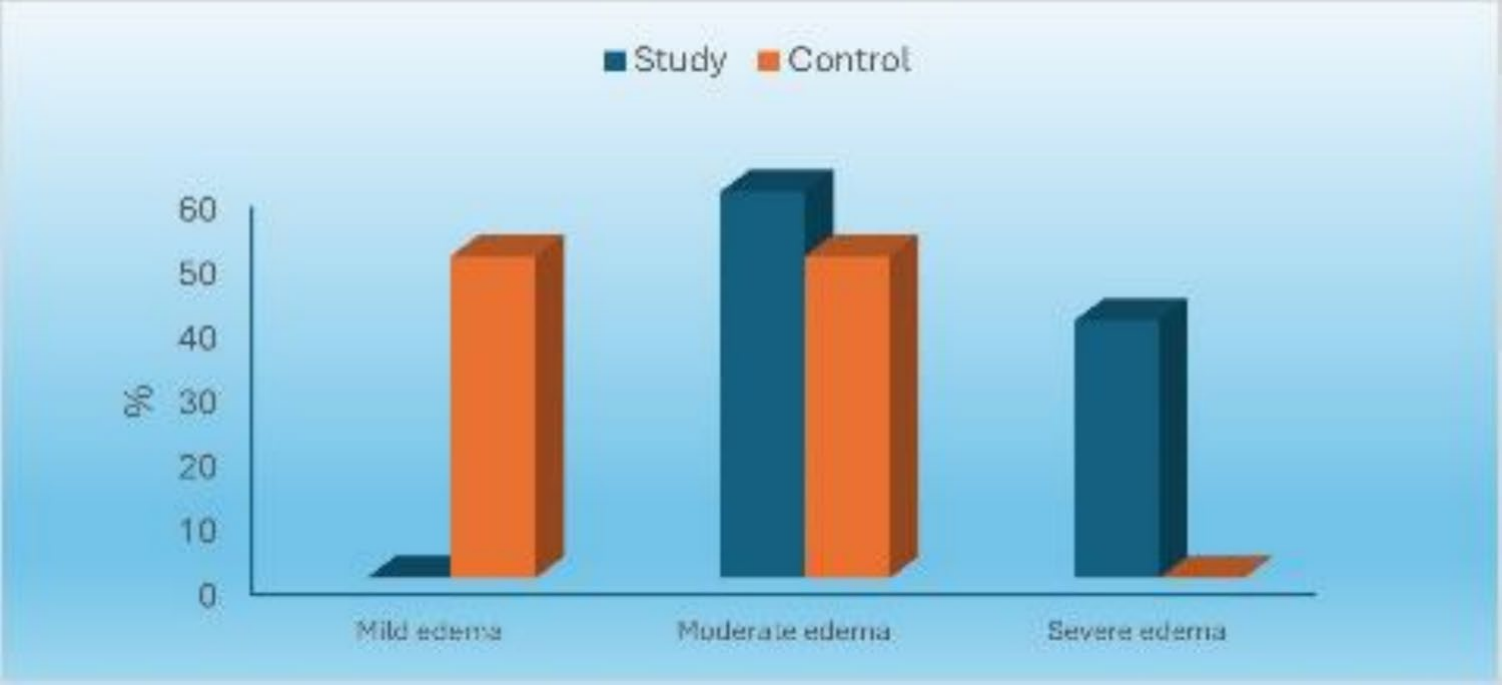
Bar chart representing severity of edema in the two groups

Study group showed statistically significantly higher prevalence of moderate and severe edema than control group which showed higher prevalence of mild edema (*P*-value < 0.001, Effect size = 0.673) [Table 6] [Fig. 19].

**Table 6:** Descriptive statistics and results of Chi-square test for comparison between severity of edema in the two groups

Severity of edema	Study (n = 15)		Control (n = 15)		P-value	Effect size (v)
	n	%	n	%		
Mild	0	0	7	46.7	< 0.001*	0.673
Moderate	9	60	8	53.3		
Severe	6	40	0	0		

^*^: Significant at P ≤ 0.05

#### Implant stability assessment using Osstell (ISQ)

### Comparison between the two groups

Whether immediately post-operative or at second stage, there was no statistically significant difference between ISQ scores in the two groups (*P*-value = 0.067, Effect size = 0.115) and (*P*-value = 0.176, Effect size = 0.064), respectively.

### Changes within each group

In the study group, there was a statistically significant increase in ISQ scores at the second stage (*P*-value = 0.013, Effect size = 0.202).

In control group, there was no statistically significant change in ISQ scores at the second stage (*P*-value = 0.128, Effect size = 0.081) [Table 7].

**Table 7:** Mean, standard deviation (SD) values and results of repeated measures ANOVA test for comparison between ISQ scores in the two groups and the changes within each group

Time	Study (n = 15)		Control (n = 15)		P-value	Effect size (Partial Eta squared)
	Mean	SD	Mean	SD		
Immediate post-operative	69.8	9.8	75.4	5.8	0.067	0.115
Second stage	75.3	8.3	78.6	4.3	0.176	0.064
P-value	0.013*		0.128			
Effect size (Partial Eta squared)	0.202		0.081			

^*^: Significant at P ≤ 0.05

### Vertical bone gain after six months (mm)

**Fig. 20:**
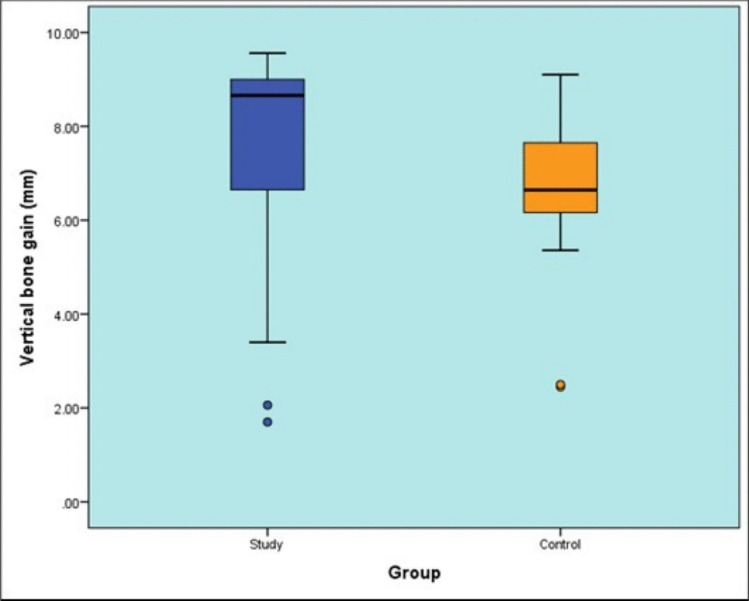
Box plot representing median and range values for vertical bone gain after six months in the two groups (Circles represent outliers)

The mean vertical bone gain was 7.25 ± 2.7 mm (guided) versus 6.5 ± 1.9 mm (freehand), with no significant difference between groups (*P*-value = 0.101, Effect size = 0.699) [Table 8] [Fig. 20].

**Table 8:** Descriptive statistics and results of Mann–Whitney U test for comparison between vertical bone gain after six months (mm) in the two groups

Study (n = 15)		Control (n = 15)		P-value	Effect size (d)
Median (Range)	Mean (SD)	Median (Range)	Mean (SD)		
8.66 (1.7, 9.56)	7.25 (2.719)	6.65 (2.45, 9.1)	6.5 (1.89)	0.101	0.699

^*^: Significant at P ≤ 0.05

## Discussion

In our study, patients were selected from among those requiring replacement for their posterior missing teeth, however suffering in this area from sinus pneumatization. Our patients could have had a single missing posterior tooth or multiple missing teeth even if they were completely edentulous in their upper arch yet necessarily fitting the criteria for sinus pneumatization.

Our sample size calculation was based on the number of implants rather than the number of sinuses because each implant was going to have its independent outcome measurements such as RFA. So, each implant counted for itself as a sample rather than the number of sinuses.

While our randomized design provides robust internal validity, the modest sample size (n = 15 per group) limits subgroup analyses and detection of smaller effect sizes. Post-hoc power analysis indicates we could only identify differences > 35% in complication rates (power = 0.8, α = 0.05). This underscores the need for cautious interpretation of non-significant findings, particularly regarding rare complications like membrane perforation (observed in 1 vs. 2 cases). Larger multicenter studies are warranted to confirm these preliminary findings.

The patient’s age had to be above the age of 18 years for two reasons, firstly it would’ve been very difficult to have sinus pneumatization below that age [30], and secondly to have reached the adult chronological age whereby a dental implant and a fixed restoration may be delivered to the patient [31]. We had no upper limit of age since this was the recommendation of the royal college of surgeons in their guidelines concerning dental implant placement. They advise that the patient can tolerate the treatment without the risk of additional factors complicating the treatment [32].

In our study, patients underwent an open approach for maxillary sinus elevation. So, our selection criteria for having the bone height to be at least 4 mm was in accordance with Anson and Horowitz in 2013, where they proved success of osteotome sinus floor elevation in bone height of 4 mm or less [33]. While at the same time a 7 mm residual bone height was the maximum height chosen in our study to allow for lateral sinus augmentation, since lateral sinus augmentation is not recommended when the bone height is more than 7 mm according to the classical Carl E. Misch’s Classification [34].

Periodontal therapy was performed whenever necessary pre-operatively also to ensure the eligibility of the site and rule out any variables that may result due to periodontal inflammation. Heavy smokers who smoked more than ten cigarettes per day were not accepted to participate in our study until a smoking reduction/cessation protocol was offered for them and they accepted it [35].

From our exclusion criteria were patients who had active infections related to the site to be augmented or implanted as well as active periodontal infections or active sinus infections. This was to eliminate any cause of bacterial infection that might’ve in turn affected the outcomes of implant survival rate [36].

Patients with parafunctional habits were also considered a relative contraindication to dental implant placement and restoration due to the undue overload on the prosthesis which may be transferred to the implants and lead to its failure as posterior maxilla already have low density and deficient height, therefore unfavorable conditions may complicate the treatment [37].

Our study design was a randomized controlled clinical trial because the patients were randomly selected from those patients attending the outpatient’s clinics of Suez Canal University as well as our patients were randomly allocated to either the test or control group using randomizing software (Random.org).

Our sample type was convenient sample because our patients were selected from those attending the faculty of dentistry outpatient clinic and we can only assume it represents the population as much as possible however it was based on the convenience of those patients attending to our facility. However, choosing a true random sample from society would have been extremely difficult in our project and requires having a complete population sampling frame, random sample from the sampling frame, and no attrition which means that everyone that was selected representing the whole of the population of our society participates in the study [38].

Our study was a double blinded study since both the statistician and the outcome assessor were blinded. The statistician was sent the results raw data in the form of numbers inside an excel sheet related to “group 1” and “group 2”. He was not informed which group was the test and which was the control. However, the surgeon and the main author of this study kept it in his records for reference. The outcome assessor was blinded upon taking the measurements and recording the outcomes from the patients, he did not know which patient was allocated to the test or the control groups. However, the surgeon performing the procedures could not be blinded because of the use of the stereolithographic surgical guide in the study group and therefore the surgeon had to be aware of it.

To ensure randomization and random allocation, a random sequence had to be generated. The choice of the electronic software, namely “Random.org” was made to ensure equal distribution of the patients to the two groups. To ensure allocation concealment to avoid selection bias, the operator or the surgeon was not informed of that generated sequence. But it was kept in a sealed envelope with the outcome assessor who was independent from the surgeon and every time after a patient was given a turn based on his time to enter the study, a number was given to him based on his turn and only after that the allocation was revealed to the surgeon to be able to perform the procedure. All the above measures were employed to minimize the risk of bias in our study as far as possible.

Pre-operative CBCT scans were performed for all patients to ensure their eligibility and therefore enrolled in our study as well as to determine the length and width of the residual bone. This was aimed at properly determining the location of the window as well as preparing the drilling lengths in the test group [39].

The utilized implant type in the current study offered cutting threads which aided in having enhanced primary stability for implant placement in the already low residual bone height [40]. Also, it was an implant that offered platform switching at the implant abutment connection which was necessary to ensure stability of the marginal bone as it shifts the component of crestal bone resorption from vertical to horizontal towards the platform switch and this is in fact is very desirable in maxillary sinus augmentation specially to maintain the biological width formed at the time of loading stable without further deterioration and loss of vertical bone height..

The implant size used in all cases had to be standardized to avoid any confounding variables. And the choice of diameter was set to 4.3 mm to suit both premolar and molar teeth. In addition, its length was set to 11.5 mm to ensure satisfactory implant extension into the sinus cavity to ensure sufficient measurements of bone height thereafter. It was found that in the literature using this length, also referred to as “standard implant”, showed a high success rate ranging from 94%−100% [41].

One of the chronic problems of the conventional surgical guide has been its massive volume. It requires not only sufficient interach space but also huge reflection of the flap. The excessive reflection of the flap is responsible for the long operation time, possibility of nerve injury such as infraorbital nerve, post-operative swelling, and discomfort [24].

In our study we adjusted the surgical guide to reduce its volume by containing only the essential information. The mesial, distal, and inferior boundaries of the lateral window play a fundamental role in a rational consideration for determination of the position of the lateral window. Moreover, the surgical guide also entails the estimated location of the superior margin since the cutting edge traces the inferior half of the lateral window [25].

In actual surgery, a surgeon’s field of vision often varies according to his/her standing point. It is further obstructed due to the mesial dentition or buccal mucosa [42]. The guide was fixed in place by anchor pins for stability. Although it succeeded in achieving immobility during the surgery, interference with the path of implant, additional bone removal from anchor pin and demand for solid bone quality remain as its limitations. Moreover, the inconvenience in withdrawing and adapting the guide during the surgery is a major drawback since it is recommended to remove the guide by elevating the sinus floor. On the other hand, the tooth-borne surgical guide ensures favorable stability with more convenience [43].

According to [43] one of the major advantages of the computer aided designing of the lateral window was the ability to identify and avoid cutting through the maxillary sinus septae by modifying the window design, which subsequently reduces the operating time and minimize possibility of membrane perforation and allow the utilization of the cortical septum in providing more support to the placed implants. In addition to that, in some cases the operator could place the implant engaging the cortical septum to enhance the primary stability of the implant. That was contrary to our results, which showed no statistically significant difference in surgical time, presence of intraoperative complications and primary stability of implants between both groups.

Perforations of the Schneiderian membrane are the most frequent intraoperative complication of lateral maxillary sinus lift for sinus elevation. The reported incident rate ranged from 10 to 60%. Out of thirty cases in our study, three suffered from membrane perforations, in accordance with the literature [44] systematically reviewed the incidence of membrane perforations in patients with lateral maxillary sinus lift. In this review, twelve studies and 388 membrane perforations were included. The incidence of membrane perforations ranged from 3.6% to 41.8%, leading to a weighted prevalence of 23.5% [45]

A recent systematic review by Younes et al. in 2018 [46] analyzed eleven studies about patient related outcomes after lateral sinus floor elevation. Unfortunately, due to the high heterogeneity in study design (1 cohort study; one retrospective case series; two prospective case series; seven randomized controlled trials), graft materials and evaluation of outcome variables, only a descriptive data analysis was provided. The review included studies in which either uni-lateral, or bi-lateral or both sinuses lift procedures were performed. The number of subjects included in most of the studies was generally very low: nine studies enrolled less than 40 patients. Data regarding pain was reported in eight of the eleven studies, using a 0–10 or 0–100 VAS, or 3- to 5-point scales. Pain was evaluated for 7 days in all studies, except for [47] (4 days), [48] (day of the surgery, 1 st and 7 th postsurgical day), and [49] first week and 14 th day). Edema was documented in only four studies [50–53]. An Oral Health Impact Profile-14 (OHIP-14) was used in one study to assess patient satisfaction [47].

In our study there was no statistical significance between both groups except after two days, where the study group showed statistically significantly lower pain score than control group. But there were changes within each group. In the study group, there was a statistically significant change in pain scores by time to seven as well as seven to eight days, there was a statistically significant decrease in pain scores. From eight to nine as well as nine to 10 days, there was no statistically significant change in pain scores. As regards control group, there was a statistically significant change in pain scores by time. Pair-wise comparisons between time periods revealed that there was no statistically significant change in pain scores after two days followed by a statistically significant decrease in pain scores from two to three, three to four, four to five, five to six, six to seven as well as seven to eight days. There was no statistically significant change in pain scores from eight to nine as well as nine to 10 days.

That was like what was observed by Mardinger et al. in 2009 [51]. These findings are in line with previous studies that showed a pain peak on the day of surgery or first post-operative day followed by a gradual decrease in the following days [46, 50–53]

Regrettably, swelling was the most evident sign (97.36% of patients) [46]. We must point out that this parameter is difficult to assess in a quantitative or qualitative way, and it is assessed subjectively by the patient. We found an article that attempted a quantitative evaluation through an optical 3-D imaging analysis for facial volumetric changes [48]. In another study, the distance between the gonion and the external canthus of the eye was measured [47].

In our study facial measurements were obtained using a measuring tape while the patient was sitting upright, and the mandible was in the physiological rest position. Five points on the face were used: most posterior point at midline on tragus (A), lateral canthus of eye (B), most lateral point on corner of mouth (C), soft tissue pogonium, which is the most prominent point at midline on chin (D), and most inferior point on the angle of the mandible (E). Measurements were taken preoperative and on the third day post-operatively (day 3), the facial swelling was evaluated by measuring the same three lines of day 1 (A to C), (B to E), and (A – D) as described by Nedal in 2015 [29]. In our study the study group showed statistically significantly higher score than the control group.

The severity of edema was also evaluated using VAS where the study group showed a statistically significantly higher prevalence of moderate and severe edema than control group which showed higher prevalence of mild edema. That was attributed to a larger flap design to accommodate the size of the stereolithographic guide and the more surgical time that was used.

Our observations regarding swelling were comparable to previous research. Fabbro et al. in 2015 observed the highest level of swelling on the first post-surgical day [53], with a gradual decline until the seventh day. Delilbasi et al. in 2013, observed highest values at 36 h, with a gradual decline thereafter. [52] Mardinger et al. in 2009 reported a median value of 5 (on a 0–5 VRS) on the second day after surgery, with a decline until the seventh day on which 21% of patients still reported swelling [51]. Farina et al. in 2018 observed a peak on days 1 and 2 with a gradual decline up to the seventh day [49].

Implant stability was measured immediately following insertion of implants and 6 months postoperatively before loading of the abutments using the OsstellISQ® device. The mean ISQ value recorded 6 months postoperatively (at second stage) increased significantly. Comparable results were obtained by Abd-Almonem, Soliman, and Shokry in 2022 and Marković et al. in 2016 [54, 55], where the mean ISQ values significantly increased 6 months after sinus lifting. According to the literature, ISQ numbers greater than seventy designate high implant stability and success of the implant placed [55].

Vertical bone height has been assessed preoperatively and after 6 months postoperatively. A statistically significant increase in bone height results agree with Arora et al. in 2019 [56] as average vertical bone height significantly increased 6 months postoperatively in the evaluation of sinus floor augmentation. However, there was no statistically significant difference between both groups in our study. The comparable vertical bone gain between groups may reflect the inherent biological limitations of the sinus augmentation technique itself, where final bone dimensions are more dependent on healing factors than initial graft positioning. This suggests that while guided surgery offers technical advantages, it may not necessarily translate to greater bone volume in straightforward cases [57].

The equivalent complication rates and bone gain between groups may reflect two factors: the technical proficiency of surgeons in both arms (minimizing freehand errors), and the biological'ceiling effect'of sinus augmentation where ideal graft placement whether guided or manual achieves similar osteogenic potential [58, 59]. This aligns with recent meta-analyses showing guided surgery's primary benefit may be operative time reduction rather than complication avoidance in routine cases [60].

## Conclusion

This randomized controlled trial found no statistically significant differences in complication rates or vertical bone gain between stereolithographic-guided and freehand lateral sinus lift procedures, suggesting comparable efficacy for routine cases. While the precision of guided surgery offers technical advantages, our results indicate that surgeon experience and biological healing factors may equalize outcomes in straight forward anatomies.

## Study limitations

However, the study’s sample size (n = 15/group) limits detection of subtle differences, particularly for rare complications such as presence of septae and blood vessels anastomosis. Larger multicenter trials with longer follow-up are warranted to validate these findings and investigate potential benefits of guided techniques in high-risk anatomies. Until then, clinicians may consider both approaches viable for uncomplicated cases, with technique selection based on operator preference and patient-specific factors.

## Data Availability

The data that support the findings of this study are available from the corresponding author upon reasonable request.
